# Deep learning‐based model for diagnosing Alzheimer's disease and tauopathies

**DOI:** 10.1111/nan.12759

**Published:** 2021-08-31

**Authors:** Shunsuke Koga, Akihiro Ikeda, Dennis W. Dickson

**Affiliations:** ^1^ Department of Neuroscience Mayo Clinic Jacksonville FL USA; ^2^ School of Medicine Osaka City University Osaka Japan

**Keywords:** Alzheimer's disease, corticobasal degeneration, machine learning, object detection, Pick's disease, progressive supranuclear palsy, random forest classifier

## Abstract

**Aims:**

This study aimed to develop a deep learning‐based model for differentiating tauopathies, including Alzheimer's disease (AD), progressive supranuclear palsy (PSP), corticobasal degeneration (CBD) and Pick's disease (PiD), based on tau‐immunostained digital slide images.

**Methods:**

We trained the YOLOv3 object detection algorithm to detect five tau lesion types: neuronal inclusions, neuritic plaques, tufted astrocytes, astrocytic plaques and coiled bodies. We used 2522 digital slide images of CP13‐immunostained slides of the motor cortex from 10 cases each of AD, PSP and CBD for training. Data augmentation was performed to increase the size of the training dataset. We next constructed random forest classifiers using the quantitative burdens of each tau lesion from motor cortex, caudate nucleus and superior frontal gyrus, ascertained from the object detection model. We split 120 cases (32 AD, 36 PSP, 31 CBD and 21 PiD) into training (90 cases) and test (30 cases) sets to train random forest classifiers.

**Results:**

The resultant random forest classifier achieved an average test score of 0.97, indicating that 29 out of 30 cases were correctly diagnosed. A validation study using hold‐out datasets of CP13‐ and AT8‐stained slides from 50 cases (10 AD, 17 PSP, 13 CBD and 10 PiD) showed >92% (without data augmentation) and >95% (with data augmentation) diagnostic accuracy in both CP13‐ and AT8‐stained slides.

**Conclusion:**

Our diagnostic model trained with CP13 also works for AT8; therefore, our diagnostic tool can be potentially used by other investigators and may assist medical decision‐making in neuropathological diagnoses of tauopathies.

## Graphical abstract

We developed a deep learning‐based tool for diagnosing Alzheimer’s disease (AD), progressive supranuclear palsy (PSP), corticobasal degeneration (CBD) and Pick’s disease (PiD), based on tau‐immunostained digital slide images. Combining an object detection model that could recognize and count five tau lesion types and a random forest classifier could successfully differentiate four tauopathies. A validation study using hold‐out datasets of CP13‐ and AT8‐stained slides from 50 cases showed >95% diagnostic accuracy in both CP13‐ and AT8‐stained slides.

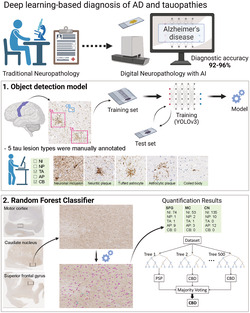

**Key points**

- We developed an object detection model that could recognise and count five different tau lesion types (neuronal inclusions, coiled bodies, tufted astrocytes, astrocytic plaques and neuritic plaques) from digitised images of tau immunostained slides.
- Using quantitative tau lesion burden from the object detection model, we generated a random forest classifier to recognise distinct subtypes of tauopathies.
- The random forest classifier could differentiate Alzheimer’s disease, progressive supranuclear palsy, corticobasal degeneration and Pick’s disease with high diagnostic accuracy.
- The results suggest that machine learning methods can be applied to facilitate the differential diagnosis of uncommon neurodegenerative tauopathies.

## INTRODUCTION

As society ages, the number of patients with neurodegenerative disorders presenting with dementia or movement disorders will increase.^1^ Tauopathy is one of the major categories of neurodegenerative disorders, characterised by an accumulation of abnormal tau protein in neurons and glia accompanied by neurodegeneration.^1^ Alzheimer's disease (AD), the most common secondary tauopathy, is a leading cause of dementia globally.^2^ Patients with progressive supranuclear palsy (PSP) and corticobasal degeneration (CBD) present with movement disorders and dementia, and Pick's disease (PiD) is most often characterised by behavioural problems and dementia.^1^ Although the advent of imaging and fluid biomarkers for tauopathies is potentially promising,^3^, ^4^, ^5^ pathological assessment at autopsy remains the gold standard for final diagnosis.

Pathologic diagnostic criteria have been proposed for these disorders^6^, ^7^, ^8^, ^9^; however, some challenges need to be addressed. First, neuropathological diagnosis is a time‐consuming process that requires highly trained experts.^10^, ^11^ Second, inter‐ and intra‐rater variabilities between observers are unavoidable.^4^, ^12^, ^13^ Third, the number of pathologists has decreased globally.^14^ The increasing number of patients with neurodegenerative disorders makes it critical to have scalable, cost‐effective means of post‐mortem diagnoses that can compensate for the decreasing number of neuropathologists.^14^

Digital pathology and machine learning‐based approaches have recently been introduced into the field of pathology.^10^ These methods hold great promise to improve the reproducibility of pathologic diagnosis.^10^ Deep learning is a subfield of machine learning, which has been used in image classification and object detection in anatomic pathology.^15^ In neuropathology, a deep learning‐based model that was able to detect and quantify neurofibrillary tangles (NFTs) in AD and other tauopathies has been reported.^16^ Other investigators developed and validated deep learning‐based models that could identify amyloid plaques and cerebral amyloid angiopathy in AD.^11^, ^17^ Deep learning‐based image classification was able to differentiate tufted astrocytes in PSP, astrocytic plaques in CBD and neuritic plaques in AD with 99% precision and recall.^18^ Although these deep learning‐based approaches are promising ways to reduce the burden on neuropathologists in terms of lesion identification and quantification, the interpretation of the quantified data to generate a diagnosis still requires a pathologist. A model that can interpret quantitative data and make a diagnosis is needed to assist this most crucial task.

We previously reported a decision tree classifier for differentiating PSP and CBD with 99% accuracy.^19^ This classifier was created using the semi‐quantitative tau lesion burdens (i.e., NFT, astrocytic inclusions [tufted astrocytes in PSP and astrocytic plaques in CBD], coiled bodies and tau threads) in vulnerable brain regions in PSP and CBD (i.e., caudate nucleus, red nucleus and motor cortex).^19^ On the basis of this finding, we hypothesised that using the quantitative tau burden ascertained from a deep learning‐based object detection model can achieve a more accurate, objective and reproducible diagnosis and that it can be applied for a wider range of tauopathies in addition to PSP and CBD.

**FIGURE 1:**
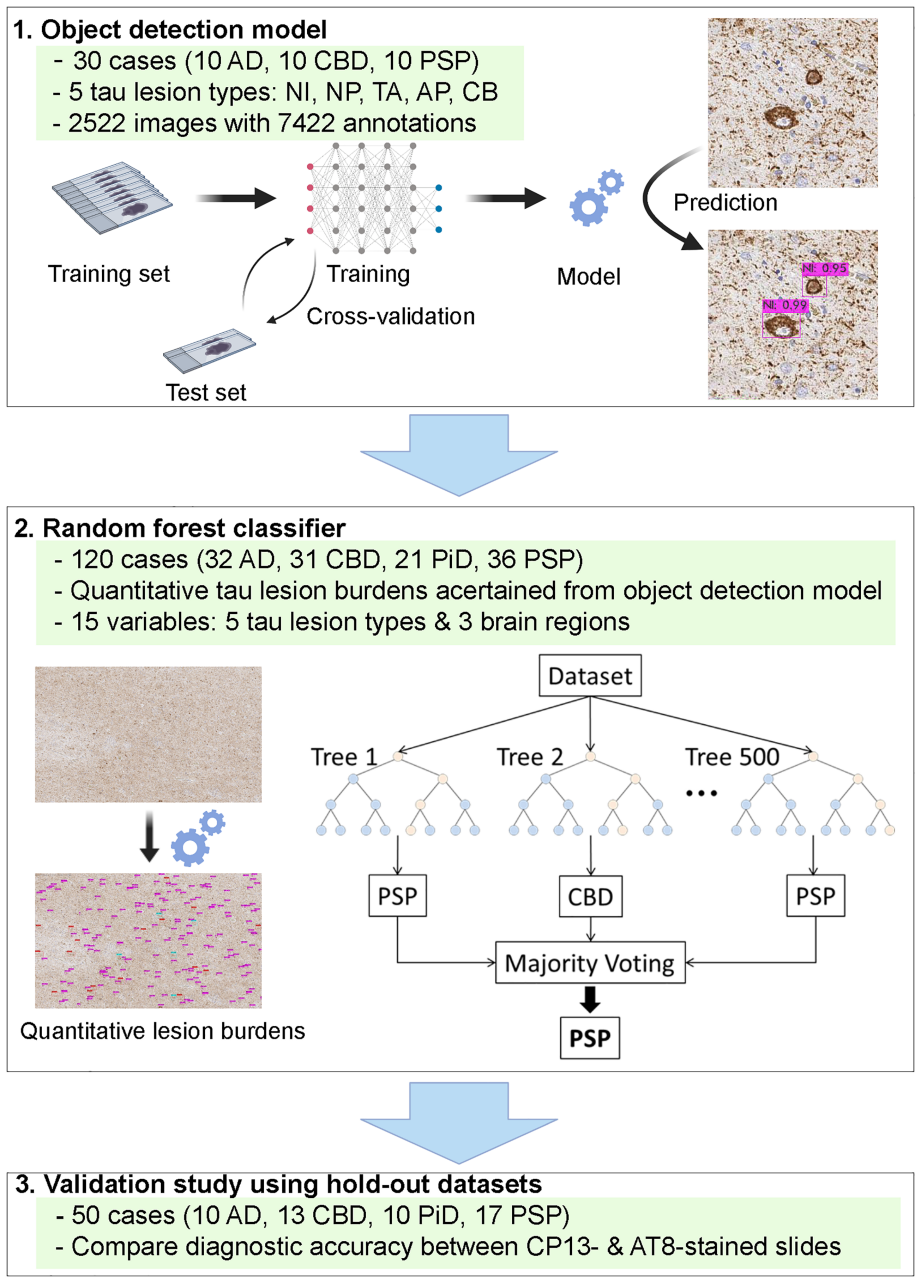
Overview of the diagnostic model consisting of object detection model and random forest classifier. Thirty cases are used for training the object detection model. One hundred and twenty cases are used to construct random forest classifier. A validation study is performed using 50 cases in hold‐out datasets. Both CP13‐stained slides and AT8‐stained slides are used. Abbreviations: AD, Alzheimer's disease; AP, astrocytic plaques; CB, coiled bodies; CBD, corticobasal degeneration; NI, neuronal inclusions; NP, neuritic plaques; PiD, Pick's disease; PSP, progressive supranuclear palsy; TA, tufted astrocytes

In the present study, we developed a deep learning‐based tool for differentiating multiple tauopathies from tau‐immunostained digital slide images. This tool consists of two models—(1) object detection and (2) random forest classifier (Figure 1). The object detection model identified and quantified five representative tau lesions in regions of interest. Using the quantitative tau burden in multiple brain regions, we constructed random forest classifiers that could differentiate AD, PSP, CBD and PiD. Our diagnostic model was trained with CP13‐immunostained slides, but it also achieved high diagnostic accuracy in AT8‐immunostained slides in a hold‐out dataset. To the best of our knowledge, the present study constitutes one of the earliest reports of end‐to‐end machine learning‐assisted diagnostic systems in the field of neuropathology.

## MATERIALS AND METHODS

### Case selection and ethical approval

All brain tissues used in this study were from the Mayo Clinic brain bank for neurodegenerative disorders. For developing an object detection model, a total of 30 cases (10 cases each of AD, PSP and CBD) from the Mayo Clinic brain bank were used. For constructing decision tree and random forest classifiers, 120 cases (32 AD, 36 PSP, 31 CBD and 21 PiD) were used. Of those, 8 PSP and 3 CBD cases had a concurrent neuropathologic diagnosis of AD. A hold‐out dataset consisting of 50 cases (10 AD, 17 PSP, 13 CBD and 10 PiD) was used for the validation study. Of those, 7 PSP and 4 CBD cases had a concurrent neuropathologic diagnosis of AD. The demographic and clinicopathologic data of all cases in this study are provided in Tables S1–S3. Brain autopsies were performed after consent of the legal next‐of‐kin or individuals with legal authority to grant permission for autopsy. De‐identified studies of autopsy samples are considered exempt from human subject research by the Mayo Clinic Institutional Review Board.

### General pathological evaluation

Formalin‐fixed brains underwent systematic and standardised sampling with neuropathologic evaluation by a single, experienced neuropathologist (DWD). Paraffin‐embedded 5‐μm thick sections mounted on glass slides are stained with haematoxylin and eosin and assessed with thioflavin S fluorescent microscopy. NFTs and senile plaques were quantified using thioflavin S fluorescent microscopy in association cortices (frontal, temporal and parietal), primary cortices (visual and motor), hippocampus (CA1, CA4 and subiculum) and adjacent cortex, amygdala, basal ganglia and cerebellum.^20^, ^21^ Pathological diagnosis of AD was according to published criteria.^8^ Immunohistochemistry for phospho‐tau (CP13, mouse monoclonal; 1:1000) was performed to establish a pathologic diagnosis of PSP, CBD and PiD.^6^, ^7^, ^13^, ^22^, ^23^ Gallyas staining, as well as immunohistochemistry for 3‐repeat (RD3, Millipore, Temecula, CA) and 4‐repeat (RD4, Millipore) tau, was performed to assist the diagnosis of PiD. All immunohistochemistry was done using an IHC Autostainer (Thermo Fisher Scientific, Waltham, MA, USA), DAKO EnVision™+ reagents (Dako) and 3,3‐diaminobenzidine as the chromogen. Immunostained slides were counterstained with haematoxylin and cover‐slipped.

### Annotation of tau lesions using digital slide images

All immunostained sections were scanned at ×20 magnification on the ScanScopeXT (Aperio Technologies, Vista, CA) to obtain whole slide digital JPEG images. A total of 2522 images (500 × 500 pixels = 6313 μm^2^) were taken from 10 cases each of AD, PSP and CBD. The five tau lesions, neuronal inclusions, tufted astrocytes, astrocytic plaques, coiled bodies and neuritic plaques, were annotated manually by an investigator (SK) using LabelImg, a graphical image annotation tool written in Python. The coordinates of bounding boxes and object classes were obtained and saved as text files. All images and text files were compressed as a zip file and uploaded to Google Drive.

### Training object detection model

YOLOv3 is an open‐source object detection architecture.^24^, ^25^ The backbone network of YOLOv3 is Darknet‐53, which includes 53 convolutional layers and uses skip connections to avoid vanishing gradient. The network is trained using images of different scales. During training, the network randomly resizes input images from 320 × 320 pixels to 608 × 608 pixels. In the prediction stage, the network uses three scale feature maps, where small feature maps provide semantic information and large feature maps provide more accurate information. There have been many studies that have used YOLOv3 detector, such as detecting dermatologic lesions (e.g., melanoma and benign nevi) in dermoscopic images^26^ and classifying leukocytes on digital images of peripheral blood smears.^27^

In the present study, we used the YOLOv3 model for developing an object detection model that could detect various tau lesion types on CP13‐immunostained slides. All images were randomly divided into training and test sets with the ratio 4:1 in Models 1 and 2 and 1:1 in Model 3. For training, hyper‐parameters were defined as follows: intersection over union = 0.50; batch size = 128; subdivisions = 32; and maximum iterations = 10,000. For evaluating the model, precision, recall, average precision for each class (e.g., tufted astrocytes and astrocytic plaques) and the mean average precision (mAP) were calculated. The average precision was defined as the mean of the precision values at these chosen 11 recall values (i.e., 0, 0.1, 0.2, 0.3, 0.4, 0.5, 0.6, 0.7, 0.8, 0.9 and 1.0).

$$\text{Precision}=\frac{T\mathrm{rue}\;\text{positive}}{T\mathrm{rue}\;\text{positive}+F\text{alse positive}}.$$

$$\text{Recall}=\frac{T\mathrm{rue}\;\text{positive}}{T\mathrm{rue}\;\text{positive}+F\text{alse negative}}.$$

$$\mathrm{mAP}=\frac{1}{n}\sum_{k=1}^{k=n}\mathrm{APk}.$$

In the equation, *n* refers to the number of classes, and *AP*_*k*_ refers to the average precision of class *k*. We selected the optimal weight based on the mAP. Training loss and mAP during training are provided in Figures S1–S3. One GPU (Tesla T4, NVIDIA), which has 2560 CUDA cores, provided by Google Colaboratory, was used for training. The training processes were performed with our Python code on Google Colaboratory platform.

### Data augmentation

To increase the size of the training dataset, we performed data augmentation by rotating images at 90°, 180° and 270°.^28^ We generated two augmented datasets by using different ratio of training/test dataset. For the first augmented dataset (Model 2), 2522 images were split into training and test sets at 4:1. Subsequently, 2018 images in the training set were augmented to 8072 images. For the second augmented dataset (Model 3), 2522 images were split into training and test sets at 1:1. Subsequently, 1261 images in the training set were augmented to 5044 images. All manipulations were performed with our Python code on the Jupyter Notebook (version 6.0.3).

### Tau burden quantification

A test image (5000 × 3000 pixels) was captured per case from the motor cortex, grey matter of superior frontal gyrus and caudate nucleus. All digital slide images were opened in Aperio ImageScope, and regions of interest were selected where the burden of pathology was greatest. In total, 360 test images (3 brain regions in 120 cases) were obtained and uploaded to the Google Drive. All 360 images were processed to obtain a quantitative tau burden for each tau lesion using the object detection model that we developed. Test images were split into 60 small tiles (500 × 500 pixels), and each tile was processed by object detection. After processing, 60 tiles were combined and given as original test images with bounding boxes and predicted labels (e.g., neuronal inclusions). The total number of each tau lesion was calculated in each test image.

### Construction of decision tree and random forest classifiers

Decision tree classifiers were created using the ‘Scikit‐Learn’ Python module.^29^ Classification and regression tree models and Gini impurity measures were used to construct decision trees. A total of 120 cases (32 AD, 31 CBD, 21 PiD and 36 PSP) were divided into a training set (90 cases) and a test set (30 cases) using the ‘random‐state’ function 30 times. We calculated the average training and test scores in these 30 random sets. The target variables were the pathological diagnoses, and the dependent variables were the quantitative tau burdens in three brain regions (i.e., motor cortex, superior frontal gyrus and caudate nucleus). The quantitative burdens of each tau lesion type were ascertained from the object detection model. We generated random forest classifiers using 500 decision tree classifiers. The average training and test scores were calculated in 30 different random datasets to evaluate the diagnostic performance.

### Validation study using hold‐out datasets

We performed a validation study using two hold‐out datasets. The first hold‐out dataset included 150 CP13‐stained slides from three brain regions of 50 cases (10 AD, 17 PSP, 13 CBD and 10 PiD). The second hold‐out dataset consisted of 150 AT8‐stained slides (mouse monoclonal; 1:2500, Dako, Carpinteria, CA, USA) from the same 50 cases. Tau lesion burdens were quantified, and we applied the random forest classifier to predict the pathological diagnoses of 50 cases. We compared the average test scores between CP13‐ and AT8‐stained slides.

## RESULTS

### Object detection model for five tau lesion types

**FIGURE 2:**
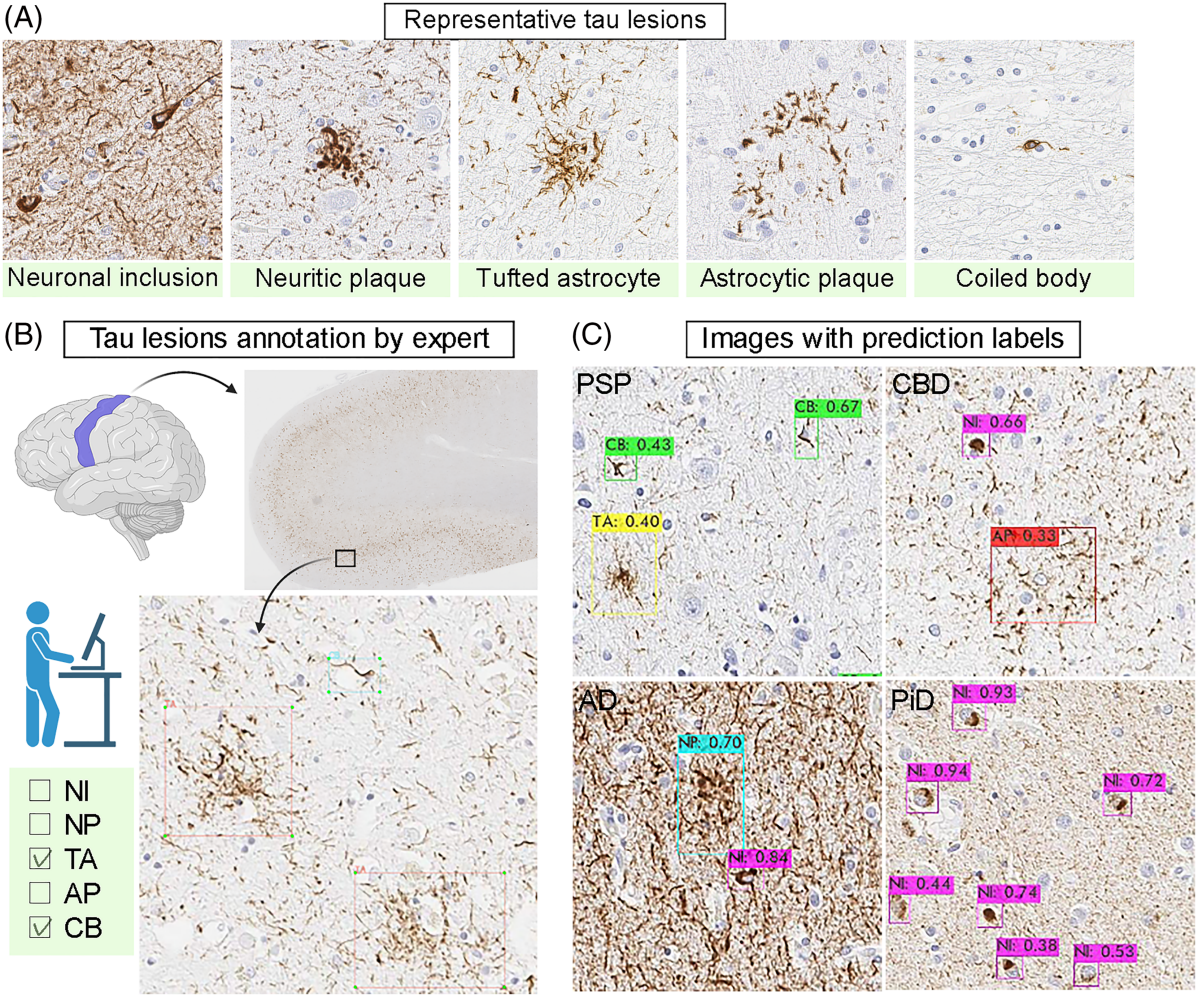
Object detection model. (A) Representative CP13‐immunostained images of five tau lesions. (B) Annotation of the representative tau lesions. A total of 2522 images are manually annotated by an investigator using LabelImg. (C) Representative results of prediction. Detected tau lesions are shown with bounding boxes with predicted labels. The number indicates the probability of each label. Abbreviations: AD, Alzheimer's disease; AP, astrocytic plaques; CB, coiled bodies; CBD, corticobasal degeneration; NI, neuronal inclusions; NP, neuritic plaques; PiD, Pick's disease; PSP, progressive supranuclear palsy; TA, tufted astrocytes

Pathological diagnosis of neurodegenerative disorders requires both macroscopic and microscopic assessment of brains at autopsy. Both the distribution and severity of neurodegeneration and tau pathologies, as well as the morphology of tau lesions, are important in the correct diagnosis of tauopathies. For example, tufted astrocytes are specific lesions in PSP, whereas astrocytic plaques are specific for CBD. On the basis of these facts, we developed an object detection model that could recognise and quantify five representative tau lesions, including neuronal inclusions, tufted astrocytes, astrocytic plaques, coiled bodies and neuritic plaques (Figure 2A). We trained the YOLOv3 model to detect tau lesions on digital images from immunostained tissues. We used 2522 images of phosphorylated‐tau (CP13) immunostained slides of motor cortex from 10 cases each of AD, PSP and CBD. The five tau lesion types were manually segmented and labelled (Figure 2B). In total, 7422 tau lesions in 2522 images were annotated. A breakdown of annotations is as follows: 3798 for neuronal inclusions, 1396 for tufted astrocytes, 1306 for coiled bodies, 469 for astrocytic plaques and 453 for neuritic plaques. All images were randomly divided into training (80%) and test (20%) datasets. We trained the model up to 10,000 iterations using these datasets, which took ~16 h. The best mAP of 66.4% was achieved at 2100 iterations (Model 1; Figure S1). The average precision of each tau lesion and mAP are shown in Table 1. The average precision was highest for neuronal inclusions (82.7%), followed by tufted astrocytes (82.5%). Figure 2C shows representative images with predicted lesion annotation. Tufted astrocytes were detected in PSP, whereas astrocytic plaques were detected in CBD. Neuritic plaques were detected in AD and detected in a subset of PSP and CBD cases. Although PiD cases were not included in the training, we applied to the object detection algorithm to CP13‐stained slides from PiD. Pick bodies were successfully detected as neuronal inclusions.

**TABLE 1:** Average precision of each tau lesion type and mean average precision

Tau lesion types	Model 1	Model 2	Model 3
Neuronal inclusions	82.7%	85.3%	85.7%
Neuritic plaques	74.8%	84.0%	68.5%
Tufted astrocytes	82.5%	85.3%	83.6%
Astrocytic plaques	42.2%	54.6%	50.6%
Coiled bodies	49.7%	62.9%	56.4%
Mean average precision	66.4%	74.4%	69.0%

### Assessment of data augmentation

**FIGURE 3:**
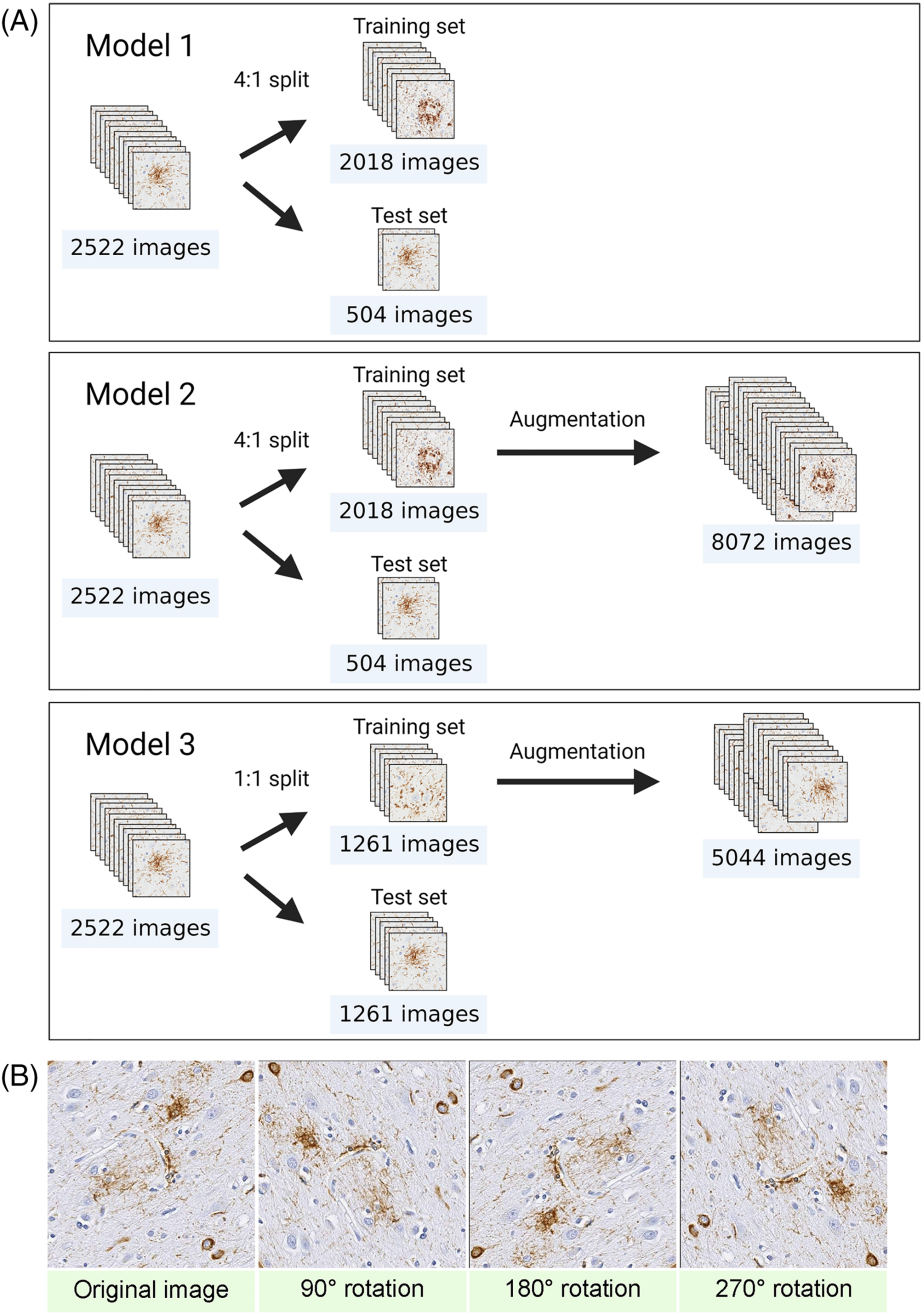
Data augmentation. (A) Flowchart of data augmentation. In Model 1, a total of 2522 images are split into training and test sets at 4:1 ratio. Data augmentation is applied to 2018 images in training set, and the number of images increases to 8072 images. In Model 2, the 2522 images are split into two sets at 1:1 ratio. Data augmentation is applied to 1261 images in training set, and the number of images increases to 5044 images. (B) Representative images of data augmentation. Images are rotated 90°, 180° and 270°

Data augmentation is a critical component of training deep learning models, which can improve the generalisation performance of image classification and object detection models.^30^ We tested whether increasing the size of the dataset by data augmentation could improve the performance of our model. We generated two augmented datasets using a different ratio of training and test datasets: 4:1 in Model 2 and 1:1 in Model 3 (Figure 3A). The data augmentation was performed by rotating images at 90°, 180° and 270° (Figure 3B). The mAP increased to 74.4% in Model 2 and 69.0% in Model 3 (Figures S2 and S3). Average precision in each tau lesion type in Models 2 and 3 is shown in Table 1.

### Tau burden quantification

**FIGURE 4:**
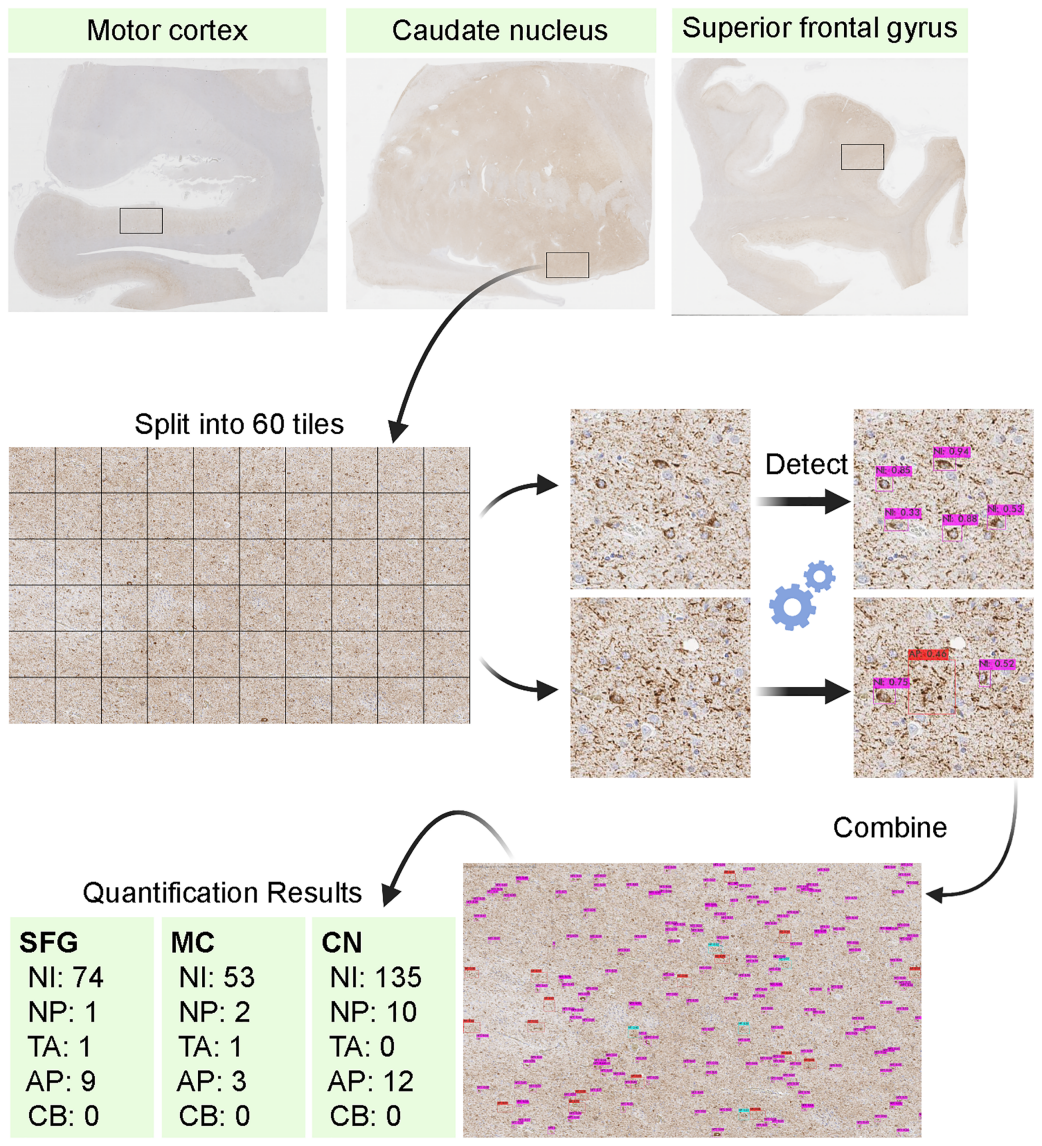
Flowchart of tau burden quantification. CP13‐stained slides from the motor cortex, caudate nucleus and superior frontal gyrus from a corticobasal degeneration (CBD) case are shown. A region of interest (5000 × 3000 pixels) is captured and split into 60 tiles. Each tile is processed for object detection, and all tiles are combined into one original image with predicted labels. Abbreviations: AP, astrocytic plaques; CB, coiled bodies; CN, caudate nucleus; MC, motor cortex; NI, neuronal inclusions; NP, neuritic plaques; SFG, superior frontal gyrus; TA, tufted astrocytes

Next, we quantified each tau lesion type in the motor cortex, superior frontal cortex and caudate nucleus from 120 cases (32 AD, 36 PSP, 31 CBD and 21 PiD). A test image (5000 × 3000 pixels) was captured from each digital whole slide image on Aperio ImageScope and saved as a JPEG file (Figure 4). In total, 360 test images (3 brain regions in 120 cases) were obtained and uploaded to the Google Drive. All 360 images were processed to obtain a quantitative tau burden for each tau lesion using the object detection model that we developed. Test images were split into 60 small tiles (500 × 500 pixels), and each tile was processed by object detection (Figure 4). After processing, 60 tiles were combined and given as original test images with bounding boxes and predicted labels (Figure 4). The total number and density of each tau lesion were calculated in each test image. The quantitative tau burdens in all cases and a representative image with bounding boxes are provided in Table S4 and Figures S4–S6.

### Decision tree classifiers

**FIGURE 5:**
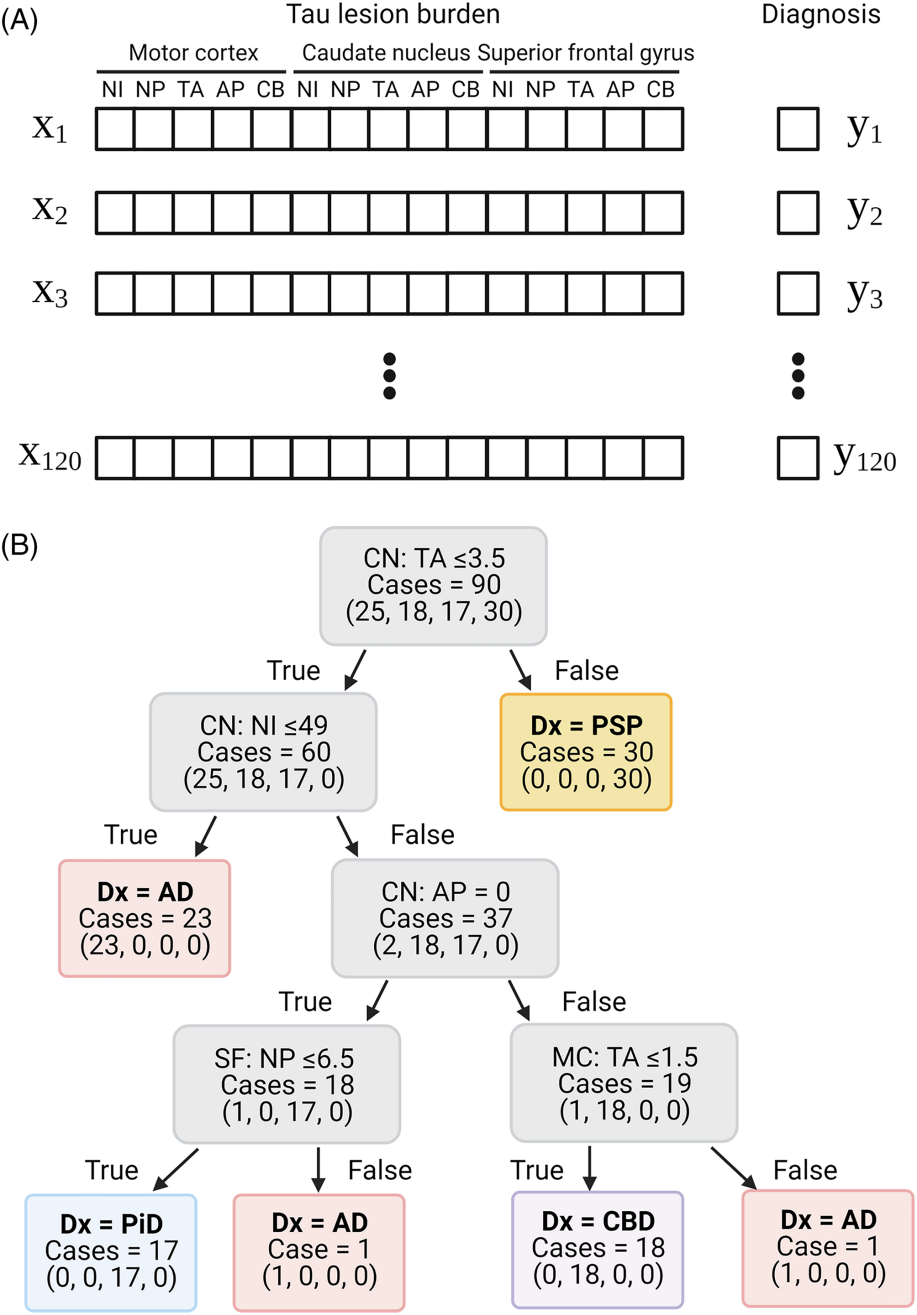
Decision tree classifier for diagnosing tauopathies. (A) Fifteen variables (*x*) and an answer label (*y*) per case are used for generating decision tree classifiers. (B) Ninety cases are used for training and creating the decision tree. All 90 cases are correctly classified using quantitative tau burden in the caudate nucleus, superior frontal gyrus and motor cortex. Values in parentheses indicate the number of AD, CBD, PiD and PSP cases, respectively. The first node asks whether the number of tufted astrocytes in the caudate nucleus is ≤3.5. If it is false, the diagnosis is PSP. If the result of first node is true, the second node asks whether the number of neuronal inclusions in the caudate nucleus is ≤49. If it is true, the diagnosis of AD is given. If it is false, the third node asks whether the number of astrocytic plaques in the caudate nucleus is = 0. If it is true, the last node asks whether the number of neuritic plaques in the superior frontal gyrus is ≤6.5. If it is true, the diagnosis of PiD is made, and if it is false, the diagnosis of AD is made. If the result of third node is false, the last node asks whether the number of tufted astrocytes in the motor cortex is ≤1.5. If it is true, the diagnosis of CBD is made, and if it is false, the diagnosis of AD is made. Abbreviations: AD, Alzheimer's disease; AP, astrocytic plaques; CBD, corticobasal degeneration; NI, neuronal inclusions; NP, neuritic plaques; PiD, Pick's disease; PSP, progressive supranuclear palsy; TA, tufted astrocytes

The quantitative data of tau burden in multiple brain regions are valuable information for diagnosing neurodegenerative diseases. The presence of tufted astrocytes and astrocytic plaques strongly suggests the diagnosis of PSP and CBD, respectively; however, the results of the object detection may include some mislabelling. To interpret and utilise the quantitative data for diagnosis, we first created decision tree classifiers using the quantitative tau burden of five lesion types in three brain regions ascertained from object detection Model 1 (Figure 5A). We randomly split 120 cases (32 AD, 31 CBD, 21 PiD and 36 PSP) into training (90 cases) and test (30 cases) sets. Figure 5B shows an example of decision tree classifiers. This decision tree correctly classified 90 training cases (training score = 1.00) and 27 out of 30 cases in the test dataset (test score = 0.97). A limitation of decision trees is their instability; they are sensitive to small variations in the training data.^31^ Different decision tree classifiers have been made even on the same training data because the training algorithm used by Scikit‐Learn is stochastic.^31^ Because the size of training and test datasets was relatively small, diagnostic accuracy in the test set was highly fluctuated by randomisation. To overcome this potential weakness, we next constructed random forest classifiers, which limit this instability by averaging predictions over multiple trees.^31^

### Random forest classifiers

**FIGURE 6:**
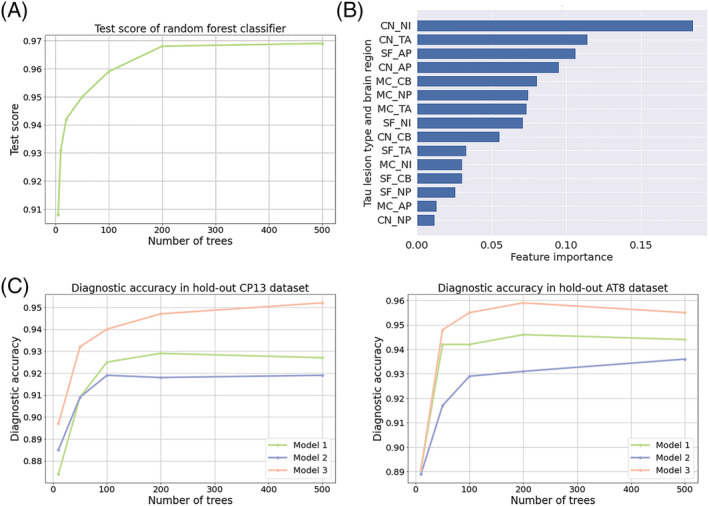
Evaluation of the random forest classifier. (A) Comparison of the test score of random forest classifiers in different number of decision trees. (B) Feature importance of the random forest classifier. (C) Diagnostic accuracy in hold‐out datasets is compared among Models 1–3 and different number of decision trees. Abbreviations: AD, Alzheimer's disease; AP, astrocytic plaques; CB, coiled bodies; CBD, corticobasal degeneration; CN, caudate nucleus; MC, motor cortex; NI, neuronal inclusions; NP, neuritic plaques; PiD, Pick's disease; PSP, progressive supranuclear palsy; SFG, superior frontal gyrus; TA, tufted astrocytes

Random forest is an ensemble learning method for classification that operates by constructing multiple decision trees during training and outputting the class by majority voting.^31^ We generated random forest classifiers based on multiple decision tree classifiers 30 times and calculated the average training and test scores. Figure 6A compares the average test scores of random forests made by a different number of decision trees. The random forest classifier achieved an average training score of 1.00 and test score of 0.96 when the number of decision trees was 500. In this classifier, the burdens of neuronal inclusions and tufted astrocytes in the caudate nucleus were the most important features for differentiating the four diseases, followed by the burden of astrocytic plaques in the superior frontal gyrus and caudate nucleus (Figure 6B **)**.

### Validation study using two hold‐out datasets

Our object detection model was trained with CP13‐immunostained slides. CP13 is not a commercial phospho‐tau antibody, and it is not widely available. AT8 is a commercial phospho‐tau antibody, which is commonly used in diagnostic neuropathology.^32^ Given the fact that the CP13 and AT8 immunostained images were almost indistinguishable (Figure S7), we hypothesised that our diagnostic model based on CP13 would also work with AT8. Addressing the question of whether the CP13‐based model can be applied to AT8‐stained slide images, we compared the diagnostic accuracy of two hold‐out datasets, CP13‐ and AT8‐stained slides from adjacent sections of 50 cases (10 AD, 17 PSP, 13 CBD and 10 PiD).

Random forest classifier using Model 1 showed an average diagnostic accuracy of 92.5% in CP13‐ and 94.6% in AT8‐stained slides. Predicted diagnoses of all cases by one of the random forest classifiers are provided in Table S1. In this test, 46 out of 50 cases (92%) were correctly diagnosed in both CP13‐ and AT8‐stained slides. In CP13‐stained slides, two CBD cases (HO‐19 and HO‐21) were diagnosed as PiD, and two PSP cases were diagnosed as AD. One of the PSP cases (HO‐42) had minimal tau pathologies in the three regions. The other PSP case (HO‐50) had concurrent Alzheimer‐type pathology (Braak NFT stage = V and Thal amyloid phase = 4). In AT8‐stained slides, one CBD case (HO‐14) was diagnosed as PiD, one PiD case (HO‐27) was diagnosed as CBD, and two PSP cases (HO‐42 and HO‐50) were diagnosed as AD. These results indicated good concurrence for the model on AT8‐immunostained slides. Quantitative tau lesion burdens in the three brain regions in all cases are provided in Tables S5 and S6.

Finally, we tested whether the random forest classifiers generated by quantitative tau burden ascertained from object detection Models 2 and 3 achieved higher diagnostic accuracy. As shown in Table 2, the average test score of the random forest using Model 2 increased to 0.976, and the diagnostic accuracy was 92.1% in CP13‐ and 93.4% in AT8‐stained slides. Random forest classifier using Model 3 showed a test score of 0.959, and diagnostic accuracy was 95.1% in CP13‐ and 95.4% in AT8‐stained slides. The comparison of diagnostic accuracy of three models with different number of decision trees is summarised in Figure 6C.

**TABLE 2:** Evaluation of random forest classifiers using different object detection models

	Model 1	Model 2	Model 3
Random forest average test score	0.965	0.976	0.959
Diagnostic accuracy in CP13 hold‐out set	0.925	0.921	0.951
Diagnostic accuracy in AT8 hold‐out set	0.946	0.934	0.954

## DISCUSSION

In the present study, we developed a deep learning‐based model that can differentiate multiple tauopathies based upon recognition of lesion types in AD, PSP, CBD and PiD. Previous methods only quantify the burden of tau pathology without attention to lesion type. We also performed a validation study using two hold‐out datasets, and they demonstrated high diagnostic accuracy (>92%) in both CP13‐ and AT8‐stained slides. These results indicate that our deep learning model trained with CP13 also works for AT8. AT8 is a widely used phospho‐tau antibody in diagnostic neuropathology; therefore, our diagnostic tool can be potentially used by other investigators. This is especially helpful for general pathologists and neuropathologists who have less experience with tauopathies.

Our diagnostic tool consists of two machine learning models—(1) object detection and (2) random forest classifier. Object detection was able to quantify each tau lesion type with high accuracy, but mislabelling of some tau lesions was unavoidable. Astrocytic plaques had relatively low mAP, probably due to the small number of annotations in training, which accounted for only 6% of annotations. Additionally, CBD usually has numerous tau‐positive threads in motor cortex, caudate nucleus and superior frontal cortex, which may hinder the detection of astrocytic plaques. Ideally, the presence of numerous tau‐positive threads should be used as a feature for CBD,^19^ but given their small size and density, it is not feasible for object detection to detect threads individually by bounding boxes. The random forest classifier makes decisions by taking into account possible mislabelling of lesions. For example, some neuritic plaques in AD may resemble astrocytic plaques and, therefore, can be mislabelled as astrocytic plaques by the object detection model. Even in such a situation, the random forest classifier would output AD rather than CBD based on other features, such as numerous neuronal inclusions. In this sense, the random forest classifier was robust for diagnosis of AD by compensating for mislabelling in the object detection model. Thus, the combination of the two models gave more accurate results than either model by themselves.

For improving the mAP of object detection model, we implemented data augmentation and improved the mAP in object detection models. Model 2 showed the highest mAP, followed by Model 3 (Table 1). The random forest classifiers based on object detection in Model 2 showed lower diagnostic accuracy in the hold‐out dataset compared with that using Model 3. This is probably because excess data augmentation may cause overfitting with the training data in Model 2. Model 3 was trained using 4:1 ratio of augmented training and test dataset (5044 images in training set and 1261 images in test set), which achieved <95% diagnostic accuracy in both CP13‐ and AT8‐stained slides. On the basis of these results, we conclude that Model 3 was the best algorithm among the three models for distinguishing AD, PSP, CBD and PiD using tau‐immunostained digital images.

Nevertheless, some cases in hold‐out datasets were misdiagnosed by our random forest classifiers. The confusion between CBD and PiD might be due to the fact that we did not use PiD cases for training in object detection model. Given the rarity of PiD, we needed to hold them out to generate random forest classifiers. Fortunately, Pick bodies were successfully detected as neuronal inclusions, even without training. As a result, both CBD and PiD cases were characterised by abundant neuronal inclusions in all three regions, particularly in the caudate nucleus (Table S4). Although the random forest classifier could diagnose the majority of PiD cases correctly, two CBD cases were misdiagnosed as PiD. Dividing the neuronal inclusion category into pretangles in CBD and Pick bodies in PiD may improve the diagnostic accuracy for these diseases. Another pitfall was noted for cases with mild tau pathology, as seen in one PSP case (HO‐42). This patient had only one tufted astrocyte in the digital image of motor cortex; thus, the random forest classifier could not recognise this case as PSP. To diagnose mild cases accurately, more regions of interest need to be analysed.

Neurodegenerative disorders in the elderly usually have multiple neuropathological processes.^33^, ^34^, ^35^ To address the question of AD co‐pathology, our cohorts included PSP with AD (PSP + AD) and CBD with AD (CBD + AD) cases in training, test and hold‐out datasets. Even having concurrent AD, our random forest classifiers could diagnose PSP or CBD correctly, except for one PSP + AD case (HO‐50). We did not define categories for PSP + AD and CBD + AD because of the limited sample size in generating random forest classifiers. Future studies should include categories of co‐pathology, such as concurrent AD, using larger sample size.

Although it is not completely knowable, it is important to discuss differences in diagnostic approach between neuropathologists and our models. Neuropathologists diagnose neurodegenerative disorders based on macroscopic and histopathologic findings, as well as clinical and genetic information, when available. For example, a neuropathologic diagnosis of AD requires assessment of both tau and amyloid‐β pathology in the neocortex, limbic and subcortical regions. In contrast, our current models predict a neuropathologic diagnosis based on only phospho‐tau immunohistochemistry in select brain regions. A decision tree classifier (Figure 5B) and feature importance of random forest classifier (Figure 6B) imply that our model make a diagnosis of AD after excluding PSP and CBD based on astrocytic tau lesions and a diagnosis of PiD based on numerous neuronal inclusions in the caudate nucleus. This counterintuitive approach may have worked in our specific experimental condition, where there are only four tauopathies in the differential diagnosis. To make our model more practical, more tauopathies and non‐tauopathies need to be added.

The value of this method for diagnosis and neuropathological research merits discussion. In neuropathological diagnosis and research, semi‐quantitative measures of pathologic lesions in four or five‐point grading scale have been commonly used.^36^, ^37^, ^38^, ^39^ Although the semi‐quantitative measurement is useful for diagnosing neurodegenerative disorders, it may inaccurately reflect the actual burden of pathology, and it has major implications in clinicopathological correlation studies using large cohorts.^34^ The object detection model will provide more objective and quantitative data of each tau lesion, rather than a measure of total tau burden. This method may be valuable in clinicopathological correlation studies, which may help identify novel clinicopathological phenotypes,^40^ as well as for correlations with molecular or genetic indices.

There are some limitations in the present study. First, our diagnostic model was trained by a single investigator with guidance from an experienced pathologist, and all immunohistochemistry was done in a single neuropathology laboratory. This may raise a concern about the generalisation performance of our model. Brain dissection and histopathological processes of each laboratory may be slightly different. Thus, digital slide images may have interlaboratory variability.^10^, ^15^, ^41^ These interlaboratory variabilities can be due to differences in the thickness of the paraffin‐embedded tissue, the reaction time of immunohistochemistry, the specific lot of the commercial antibody, the time between immunohistochemistry and scanning slides and the particular scanner that was used to capture the images.^15^, ^41^ To overcome this potential weakness, future studies need to include tissue sections with different thicknesses, slides from different laboratories and digital slide images scanned by different scanners. Second, we used CP13‐stained slides for training; however, CP13 is not a widely used antibody. AT8 is a commercial antibody detecting pSer202/pThr205, which is more commonly used in diagnostic neuropathology. Immunohistochemical images of CP13 and AT8 were almost identical, and our validation study using hold‐out datasets confirmed that the diagnostic model worked as well with AT8‐stained slides. Although the discrepancy between CP13 and AT8 was negligible, training with AT8‐stained slides might improve the diagnostic accuracy in AT8‐stained slides. Finally, our diagnostic model could differentiate AD, PSP, CBD and PiD; however, other tauopathies could also be involved in differential diagnoses. Argyrophilic grain disease is a common concurrent pathology in CBD and PSP.^42^, ^43^ Globular glial tauopathy (GGT) is a rare 4‐repeat tauopathy, characterised by globular astrocytic inclusions and globular oligocytic inclusions.^22^ The diagnosis of GGT is sometimes challenging, especially in differentiating it from PSP.^22^ Tauopathy due to *MAPT* mutations can present various types of pathology, depending on the particular mutations.^44^ Taken together, future studies need to include AT8‐stained slides for training, annotation of tau lesions in different brain regions and inclusion of more variety of tauopathies.

The purpose of our diagnostic tool was not to replace but to complement diagnostic procedures and to leverage the expertise of pathologists by improving the reproducibility of pathologic diagnoses. In the present study, our diagnostic model sufficiently differentiated AD, CBD, PiD and PSP using CP13‐stained slides, as well as AT8‐stained slides. Although the current version of our diagnostic model is not a user‐friendly graphical user interface, it is publicly accessible in our GitHub repository; therefore, other investigators will be able to use this diagnostic model. Our findings may also encourage further development of deep learning‐based diagnostic models beyond tauopathies, which can assist in decision‐making for other pathologic diagnoses. In addition to the diagnosis, the quantification of tau lesion types in our model will provide valuable information for clinicopathologic and genetic studies.

## CONFLICT OF INTEREST

The authors declare that they have no conflicts of interest.

## ETHICS STATEMENT

Brain autopsies were performed after consent of the legal next‐of‐kin or individuals with legal authority to grant permission for autopsy. De‐identified studies of autopsy samples are considered exempt from human subject research by the Mayo Clinic Institutional Review Board.

## AUTHOR CONTRIBUTIONS

Shunsuke Koga conceptualised the studies, wrote the code, performed the computational experiments, trained the models, analysed results, created figures and wrote the first draft. Shunsuke Koga also provided case selection and annotations and neuropathology data interpretation, with guidance from Dennis W. Dickson. Akihiro Ikeda wrote the code and assisted in uploading the data to GitHub. Dennis W. Dickson provided neuropathological diagnoses of all cases and provided all histopathology slides. All authors were involved in critical revisions of the manuscript and have read and approved the final draft.

### PEER REVIEW

The peer review history for this article is available at https://publons.com/publon/10.1111/nan.12759.

## Supporting information


**Table S1:** **Diagnosis results of hold‐out dataset**
Table S2: Demographic and pathologic data in 30 cases used in object detection model
Table S3: Demographic and pathologic data in 120 cases used in decision tree and random forest classifiers
Table S4: Quantitative tau lesion burdens in 120 cases for random forest classifier
Table S5: Quantitative burdens of cases in hold‐out dataset (CP13) using Model 1
Table S6: Quantitative burdens of cases in hold‐out dataset (AT8) using Model 1
Figure S1: Training loss and mAP during training in Model 1
Figure S2: Training loss and mAP during training in Model 2
Figure S3: Training loss and mAP during training in Model 3
**Figure S4: Representative image with bounding box**. The motor cortex from a patient with PSP + AD (RF‐96).
**Figure S5: Representative image with bounding box.** The caudate nucleus from a patient with PSP + AD (RF‐96).
**Figure S6: Representative image with bounding box.** The superior frontal gyrus from a patient with PSP + AD (RF‐96).
**Figure S7: Comparison between CP13‐ and AT8‐stained slides.** Representative images are taken from a patient with PSP + AD (HO‐41). Tufted astrocytes, neuritic plaques, and neuronal inclusions are present in the motor cortex, caudate nucleus, and superior frontal gyrus.

## Data Availability

The data that support the findings of this study are openly available in Zenodo at https://zenodo.org/record/5083997. All analysis code used in this study is openly available in GitHub at https://github.com/Koga-MD/DL-Tauopathies.
