# Supplementary material for: Deep learning‐based model for diagnosing Alzheimer's disease and tauopathies
Source: Neuropathol Appl Neurobiol. 2021 Aug 31;48(1):e12759. doi: 10.1111/nan.12759 (PMC9293025; doi:10.1111/nan.12759)
Supplement: Supplementary file 1 — Table S1: Diagnosis results of hold‐out dataset Table S2: Demographic and pathologic data in 30 cases used in object detection model Table S3: Demographic and pathologic data in 120 cases used in decision tree and random forest classifiers Table S4: Quantitative tau lesion burdens in 120 cases for random forest classifier Table S5: Quantitative burdens of cases in hold‐out dataset (CP13) using Model 1 Table S6: Quantitative burdens of cases in hold‐out dataset (AT8) using Model 1 Figure S1: Training loss and mAP during training in Model 1 Figure S2: Training loss and mAP during training in Model 2 Figure S3: Training loss and mAP during training in Model 3 Figure S4: Representative image with bounding box. The motor cortex from a patient with PSP + AD (RF‐96). Figure S5: Representative image with bounding box. The caudate nucleus from a patient with PSP + AD (RF‐96). Figure S6: Representative image with bounding box. The superior frontal gyrus from a patient with PSP + AD (RF‐96). Figure S7: Comparison between CP13‐ and AT8‐stained slides. Representative images are taken from a patient with PSP + AD (HO‐41). Tufted astrocytes, neuritic plaques, and neuronal inclusions are present in the motor cortex, caudate nucleus, and superior frontal gyrus. [file NAN-48-0-s001.pdf]

SUPPLEMENTARY INFORMATION

Deep learning-based model for diagnosing Alzheimer's disease and tauopathies

Shunsuke Koga, MD, PhD, Akihiro Ikeda, LLB, and Dennis W. Dickson. MD

Contents

Supplementary Appendix 1
Supplementary Table 1 2
Supplementary Table 2 3
Supplementary Table 3 4
Supplementary Table 4 7
Supplementary Table 5 10
Supplementary Table 6 11
Supplementary Figure 1 12
Supplementary Figure 2 13
Supplementary Figure 3 14
Supplementary Figure 4 15
Supplementary Figure 5 16
Supplementary Figure 6 17
Supplementary Figure 7 18

Table S1: Diagnosis results of hold-out dataset

Case ID	Disease	Age	Sex	Braak	Thal	Clinical diagnosis	Dx in CP13	Dx in AT8
**HO-1**	AD	65	F	VI	5	CBS	AD	AD
**HO-2**	AD	69	M	VI	5	CBS	AD	AD
**HO-3**	AD	70	F	VI	5	CBS	AD	AD
**HO-4**	AD	72	F	VI	5	CBS	AD	AD
**HO-5**	AD	74	F	VI	5	AD	AD	AD
**HO-6**	AD	76	F	VI	5	AD	AD	AD
**HO-7**	AD	78	M	V	5	AD	AD	AD
**HO-8**	AD	82	M	VI	5	CBS	AD	AD
**HO-9**	AD	82	M	VI	5	AD	AD	AD
**HO-10**	AD	83	F	VI	5	AD	AD	AD
**HO-11**	CBD	61	F	II	0	PSP	CBD	CBD
**HO-12**	CBD	66	M	II	3	PSP	CBD	CBD
**HO-13**	CBD	66	M	0	0	PSP v PDD	CBD	CBD
**HO-14**	CBD	67	M	I	0	PSP	CBD	**PiD**
**HO-15**	CBD	68	F	IV	5	CBS/AOS	CBD	CBD
**HO-16**	CBD	69	M	IV	0	AD v PiD	CBD	CBD
**HO-17**	CBD	69	F	III	3	MSA	CBD	CBD
**HO-18**	CBD	71	F	IV	3	PSP v CBS	CBD	CBD
**HO-19**	CBD	72	F	II	1	PSP	**PiD**	CBD
**HO-20**	CBD	74	F	II	2	FTD	CBD	CBD
**HO-21**	CBD	75	M	IV	3	CBS	**PiD**	CBD
**HO-22**	CBD	77	M	III	1	MSA	CBD	CBD
**HO-23**	CBD	82	M	IV	5	CBS	CBD	CBD
**HO-24**	PiD	53	F	0	0	bvFTD	PiD	PiD
**HO-25**	PiD	62	M	II	0	AD	PiD	PiD
**HO-26**	PiD	62	F	0	0	bvFTD	PiD	PiD
**HO-27**	PiD	63	M	IV	0	FTD/PPA v NPH	PiD	**CBD**
**HO-28**	PiD	65	M	II	0	FTD	PiD	PiD
**HO-29**	PiD	66	M	0	0	Atypical AD	PiD	PiD
**HO-30**	PiD	69	M	VI	5	Early-onset AD	PiD	PiD
**HO-31**	PiD	70	M	I	0	FTD	PiD	PiD
**HO-32**	PiD	74	F	IV	4	AD	PiD	PiD
**HO-33**	PiD	74	F	II	0	FTD	PiD	PiD
**HO-34**	PSP	64	M	III	3	PSP	PSP	PSP
**HO-35**	PSP	66	M	II	0	PSP-P	PSP	PSP
**HO-36**	PSP	67	M	IV	4	FTD v PPA	PSP	PSP
**HO-37**	PSP	68	F	II	0	PSP	PSP	PSP
**HO-38**	PSP	70	F	III	2	CBS	PSP	PSP
**HO-39**	PSP	72	M	V	5	PSP	PSP	PSP
**HO-40**	PSP	73	M	II	0	PSP	PSP	PSP
**HO-41**	PSP	74	F	VI	4	DLB	PSP	PSP
**HO-42**	PSP	75	F	II	0	PSP-PNFA	**AD**	**AD**
**HO-43**	PSP	76	M	II	0	PSP/PPA	PSP	PSP
**HO-44**	PSP	76	M	II	0	PSP	PSP	PSP
**HO-45**	PSP	77	M	I	0	PSP	PSP	PSP
**HO-46**	PSP	77	F	V	5	MSA	PSP	PSP
**HO-47**	PSP	79	F	IV	4	CBS	PSP	PSP
**HO-48**	PSP	82	M	II	0	MSA v PD	PSP	PSP
**HO-49**	PSP	83	F	V	5	PSP	PSP	PSP
**HO-50**	PSP	84	F	VI	4	PSP	**AD**	**AD**

Abbreviations: AD, Alzheimer's disease; AOS, apraxia of speech; Braak, Braak neurofibrillary tangle stage; DLB, dementia with Lewy bodies; bvFTD, behavioral variant frontotemporal dementia; CBD, corticobasal degeneration; CBS, corticobasal syndrome; FTD, frontotemporal dementia; MSA, multiple system atrophy; NPH, normal pressure hydrocephalus; PiD, Pick's disease; PPA, primary progressive aphasia; PSP, progressive supranuclear palsy; PSP-P, PSP-parkinsonism; PSP-PNFA, PSP-progressive non-fluent aphasia; Thal, Thal amyloid phase.

Table S2: Demographic and pathologic data in 30 cases used in object detection model

Case ID	Disease	Age	Sex	Braak stage	Thal phase	Clinical Diagnosis
**OD-1**	AD	56	F	VI	5	AD
**OD-2**	AD	62	F	VI	5	AD
**OD-3**	AD	66	F	VI	5	AD
**OD-4**	AD	68	M	VI	4	AD
**OD-5**	AD	69	F	VI	5	AD
**OD-6**	AD	69	M	VI	5	AD
**OD-7**	AD	73	M	VI	5	AD
**OD-8**	AD	73	M	V	5	AD
**OD-9**	AD	75	F	VI	5	AD
**OD-10**	AD	82	F	VI	5	AD
**OD-11**	CBD	60	F	II	0	CBS/AOS
**OD-12**	CBD	61	M	II	0	CBS
**OD-13**	CBD	67	M	II	0	PPA
**OD-14**	CBD	69	M	0	0	CBS
**OD-15**	CBD	69	M	III	0	FTD
**OD-16**	CBD	70	F	II	0	PSP
**OD-17**	CBD	70	M	III	0	CBS
**OD-18**	CBD	70	M	I	0	logopenic PPA
**OD-19**	CBD	72	M	III	0	CBS
**OD-20**	CBD	77	M	III	0	PSP-PAGF
**OD-21**	PSP	69	F	III	3	PSP
**OD-22**	PSP	74	F	II	0	PSP-P
**OD-23**	PSP	75	F	IV	4	CBS
**OD-24**	PSP	78	M	0	0	CBS/AOS
**OD-25**	PSP	79	F	II	0	PSP
**OD-26**	PSP	80	M	II	0	PSP
**OD-27**	PSP	81	M	0	0	PSP-P
**OD-28**	PSP	82	M	III	0	FTD
**OD-29**	PSP	84	M	IV	1	PSP
**OD-30**	PSP	84	F	0	0	PSP v MSA

Abbreviations: AD, Alzheimer's disease; AOS, apraxia of speech; CBS, corticobasal syndrome; CBD, corticobasal degeneration; FTD, frontotemporal dementia; MSA, multiple system atrophy; PPA, primary progressive aphasia; PSP, progressive supranuclear palsy; PSP-P, progressive supranuclear palsy-parkinsonism; PSP-PAGF, progressive supranuclear palsy- pure akinesia with gait freezing.

Table S3: Demographic and pathologic data in 120 cases used in decision tree and random forest classifiers

Case ID	Dx	Age	Sex	Braak stage	Thal phase	Clinical diagnosis
**RF-1**	AD	88	F	VI	4	CBS
**RF-2**	AD	86	F	VI	5	CBS
**RF-3**	AD	85	F	VI	5	AD
**RF-4**	AD	83	M	VI	5	CBS
**RF-5**	AD	82	F	VI	5	CBS v FTD
**RF-6**	AD	79	F	VI	5	AD
**RF-7**	AD	79	F	V	5	CBS
**RF-8**	AD	78	F	VI	5	AD
**RF-9**	AD	78	F	VI	5	AD/PD v CBS
**RF-10**	AD	77	M	V	5	CBS vs AD & ET
**RF-11**	AD	77	F	VI	5	CBS
**RF-12**	AD	77	F	VI	3	CBS v PSP
**RF-13**	AD	77	M	VI	5	CBS v PSP-P v AD visual variant
**RF-14**	AD	76	F	VI	5	CBS
**RF-15**	AD	75	M	VI	5	CBS
**RF-16**	AD	75	F	VI	5	CBS
**RF-17**	AD	74	M	VI	5	CBS
**RF-18**	AD	74	M	VI	5	CBS
**RF-19**	AD	70	F	VI	5	AD
**RF-20**	AD	68	F	VI	5	CBS v PCA (AD)
**RF-21**	AD	67	F	VI	5	FTD v CBS
**RF-22**	AD	67	M	VI	5	CBS (PNFA)
**RF-23**	AD	67	M	V	5	CBS
**RF-24**	AD	67	M	VI	5	CBS
**RF-25**	AD	64	F	VI	5	CBS
**RF-26**	AD	64	M	VI	5	CBS
**RF-27**	AD	63	F	VI	5	CBS
**RF-28**	AD	63	F	VI	5	CBS
**RF-29**	AD	63	F	VI	5	CBS
**RF-30**	AD	61	M	VI	5	AD
**RF-31**	AD	61	M	VI	5	CBS
**RF-32**	AD	58	F	VI	5	PCA v CBS
**RF-33**	CBD	84	F	III	0	Logopenic PPA
**RF-34**	CBD	80	F	II	2	CBS
**RF-35**	CBD	79	F	I	0	CBS
**RF-36**	CBD	78	F	II	1	CBS
**RF-37**	CBD	78	F	IV	4	CBS v PSP
**RF-38**	CBD	77	M	II	3	atypical PD v NPH
**RF-39**	CBD	76	F	III	1	CBS
**RF-40**	CBD	75	F	III	0	PSP
**RF-41**	CBD	75	M	III	0	CBS
**RF-42**	CBD	75	M	II	0	CBS
**RF-43**	CBD	74	F	III	3	CBS v atypical PSP
**RF-44**	CBD	74	F	VI	4	PSP
**RF-45**	CBD	73	M	III	0	CBS
**RF-46**	CBD	73	M	III	0	FTDP
**RF-47**	CBD	72	F	I	0	CBS
**RF-48**	CBD	72	F	II	0	CBS
**RF-49**	CBD	72	F	III	0	CBS
**RF-50**	CBD	71	F	III	3	CBS
**RF-51**	CBD	71	F	III	1	PSP v MSA
**RF-52**	CBD	71	F	II	0	FTD/PD
**RF-53**	CBD	70	F	II	1	FTD
**RF-54**	CBD	70	F	II	2	PPA
**RF-55**	CBD	69	F	II	0	CBS
**RF-56**	CBD	69	F	I	0	PSP

**RF-57**	CBD	66	M	I	0	CBS
**RF-58**	CBD	66	M	V	5	CBS
**RF-59**	CBD	65	M	II	0	CBS
**RF-60**	CBD	62	M	II	2	CBS
**RF-61**	CBD	61	F	I	0	FTD/PPA
**RF-62**	CBD	58	M	IV	0	PPA (AOS)
**RF-63**	CBD	58	M	IV	0	FTD v AD
**RF-64**	PiD	84	M	II	0	PNFA
**RF-65**	PiD	75	F	III	0	CBS
**RF-66**	PiD	74	M	0	0	FTD (SD)
**RF-67**	PiD	73	F	II	0	CBS
**RF-68**	PiD	72	F	II	1	PD v CBS
**RF-69**	PiD	72	M	0	0	PiD
**RF-70**	PiD	70	M	I	1	AD v PiD
**RF-71**	PiD	69	F	0	0	FTD v PNFA
**RF-72**	PiD	68	F	I	0	FTD
**RF-73**	PiD	68	F	0	1	PPA
**RF-74**	PiD	67	M	II	1	FTD
**RF-75**	PiD	67	M	III	3	PPA (logopenic) v AD
**RF-76**	PiD	66	M	II	2	CBS
**RF-77**	PiD	66	F	II	3	FTD v DLB
**RF-78**	PiD	65	M	II	0	FTD
**RF-79**	PiD	63	M	IV	1	AD/PD
**RF-80**	PiD	63	F	II	0	AOS
**RF-81**	PiD	61	F	I	0	FTD
**RF-82**	PiD	60	F	0	2	CBS
**RF-83**	PiD	59	M	II	2	CBS
**RF-84**	PiD	59	F	II	0	FTD
**RF-85**	PSP	91	M	II	3	PSP
**RF-86**	PSP	89	M	III	3	CBS
**RF-87**	PSP	87	F	IV	4	PSP
**RF-88**	PSP	82	F	IV	3	PSP v CBS
**RF-89**	PSP	82	M	V	4	CBS
**RF-90**	PSP	81	M	IV	3	PDD
**RF-91**	PSP	81	M	VI	5	PSP
**RF-92**	PSP	81	F	V	4	PSP
**RF-93**	PSP	80	M	V	5	PSP
**RF-94**	PSP	79	F	II	2	PSP
**RF-95**	PSP	79	M	II	3	PSP
**RF-96**	PSP	78	F	IV	5	PSP
**RF-97**	PSP	78	F	VI	4	CBS
**RF-98**	PSP	76	F	VI	5	CBS
**RF-99**	PSP	72	F	II	0	PSP v CBS
**RF-100**	PSP	72	M	I	1	PSP
**RF-101**	PSP	71	M	II	0	PSP
**RF-102**	PSP	71	M	II	0	PSP
**RF-103**	PSP	71	M	II	1	PSP
**RF-104**	PSP	71	M	III	5	MSA v PSP
**RF-105**	PSP	71	M	I	0	PSP
**RF-106**	PSP	70	M	I	0	PSP
**RF-107**	PSP	70	F	III	0	PSP v CBS
**RF-108**	PSP	69	M	II	0	PSP
**RF-109**	PSP	68	F	II	1	PSP
**RF-110**	PSP	68	F	II	0	PSP
**RF-111**	PSP	67	M	II	1	PSP
**RF-112**	PSP	67	M	II	0	PSP
**RF-113**	PSP	66	M	II	0	PSP
**RF-114**	PSP	66	F	II	2	PSP
**RF-115**	PSP	65	F	III	0	PSP

**RF-116**	PSP	65	F	II	0	PSP
**RF-117**	PSP	64	M	I	0	PSP
**RF-118**	PSP	64	F	0	0	PSP
**RF-119**	PSP	61	F	I	0	PSP-P
**RF-120**	PSP	56	M	0	0	PSP-P

Abbreviations: AD, Alzheimer's disease; AOS, apraxia of speech; CBS, corticobasal syndrome; CBD, corticobasal degeneration; FTD, frontotemporal dementia; MSA, multiple system atrophy; NPH, normal pressure hydrocephalus; PD, Parkinson disease; PDD, Parkinson disease dementia; PiD, Pick's disease; PNFA, progressive non-fluent aphasia ; PPA, primary progressive aphasia; PSP, progressive supranuclear palsy; PSP-P, progressive supranuclear palsy-parkinsonism; PSP-PAGF, progressive supranuclear palsy- pure akinesia with gait freezing.

Table S4: Quantitative tau lesion burdens in 120 cases for random forest classifier

Case ID	Dx	Motor cortex					Caudate nucleus					Superior frontal gyrus				
		TA	AP	CB	NI	NP	TA	AP	CB	NI	NP	TA	AP	CB	NI	NP
**RF-1**	AD	0	0	1	98	9	0	0	2	100	0	0	0	1	92	6
**RF-2**	AD	0	0	0	33	55	0	0	0	31	2	0	0	0	106	4
**RF-3**	AD	0	1	1	22	17	2	2	0	16	0	0	0	0	145	4
**RF-4**	AD	0	0	2	48	24	0	0	0	1	0	0	0	3	26	3
**RF-5**	AD	3	0	3	104	17	0	5	0	48	0	0	0	0	69	26
**RF-6**	AD	0	0	2	48	26	0	0	0	7	0	0	0	0	164	12
**RF-7**	AD	0	1	1	34	15	0	0	0	5	0	0	0	0	95	3
**RF-8**	AD	0	0	0	70	13	0	0	1	24	0	0	0	1	142	10
**RF-9**	AD	0	0	0	77	13	0	0	0	5	0	0	1	0	185	27
**RF-10**	AD	1	1	2	57	43	0	0	0	21	2	0	2	0	40	31
**RF-11**	AD	0	1	1	53	20	0	0	0	0	0	0	1	0	14	1
**RF-12**	AD	1	0	12	25	8	0	0	0	13	0	0	3	2	59	5
**RF-13**	AD	0	0	0	76	30	0	0	0	2	0	0	1	2	75	1
**RF-14**	AD	0	2	2	106	6	0	0	0	6	0	0	0	0	116	7
**RF-15**	AD	0	0	0	71	22	1	0	0	7	0	0	1	2	57	9
**RF-16**	AD	0	0	0	15	26	0	0	0	2	0	0	1	0	69	10
**RF-17**	AD	0	0	2	73	18	3	0	0	4	0	0	0	0	93	6
**RF-18**	AD	0	0	0	42	18	0	0	0	1	1	0	0	0	52	1
**RF-19**	AD	1	0	0	33	61	0	0	0	10	3	0	0	1	67	42
**RF-20**	AD	0	0	1	91	21	0	1	1	17	1	0	0	0	102	4
**RF-21**	AD	0	0	0	55	63	0	0	1	12	0	0	1	0	120	5
**RF-22**	AD	0	0	0	67	38	0	0	0	2	0	0	0	0	140	14
**RF-23**	AD	1	0	2	119	20	0	0	0	0	0	0	0	2	29	6
**RF-24**	AD	0	0	1	51	6	0	0	1	9	1	0	0	1	101	5
**RF-25**	AD	0	0	0	34	13	0	0	1	13	0	0	0	0	23	4
**RF-26**	AD	0	1	0	11	22	0	0	0	1	0	0	1	1	29	6
**RF-27**	AD	0	1	2	81	18	6	0	0	3	0	0	0	0	92	12
**RF-28**	AD	0	1	0	60	2	0	2	0	1	0	0	0	0	126	7
**RF-29**	AD	0	0	1	59	6	0	0	2	4	1	0	0	0	108	10
**RF-30**	AD	0	0	0	88	16	0	0	0	3	0	0	0	1	107	6
**RF-31**	AD	0	0	1	54	34	0	0	2	2	1	0	6	0	64	21
**RF-32**	AD	0	0	1	77	14	0	0	0	3	0	1	0	0	81	4
**RF-33**	CBD	0	1	1	72	0	0	9	1	69	0	0	0	0	0	0
**RF-34**	CBD	0	4	2	132	3	0	0	0	75	0	0	1	2	63	0
**RF-35**	CBD	0	0	2	36	0	0	36	0	95	2	0	12	0	144	1
**RF-36**	CBD	2	5	4	90	0	0	13	1	139	0	0	4	2	156	1
**RF-37**	CBD	0	4	7	103	6	1	14	7	117	1	6	0	3	62	1
**RF-38**	CBD	0	0	2	3	0	7	72	2	41	0	10	13	4	18	2
**RF-39**	CBD	0	0	3	97	0	0	5	0	108	0	0	3	3	112	0
**RF-40**	CBD	0	1	2	145	2	2	27	2	154	0	0	5	5	170	0
**RF-41**	CBD	0	59	4	166	6	0	25	4	217	9	0	80	3	197	3
**RF-42**	CBD	0	0	6	110	0	1	145	4	57	4	0	25	5	55	0
**RF-43**	CBD	0	0	3	68	0	0	3	0	150	0	0	15	2	103	5

**RF-44**	CBD	0	2	0	20	22	0	64	1	45	7	0	6	0	75	9
**RF-45**	CBD	0	0	2	164	1	0	27	0	82	0	0	4	1	117	0
**RF-46**	CBD	0	6	11	68	0	0	3	1	163	2	0	2	0	146	5
**RF-47**	CBD	0	32	4	88	1	0	20	1	107	0	0	7	2	159	0
**RF-48**	CBD	0	1	4	166	0	0	29	4	134	2	0	18	2	109	2
**RF-49**	CBD	0	1	0	186	8	0	18	0	128	0	0	3	4	157	1
**RF-50**	CBD	0	3	3	75	0	0	2	5	71	0	4	13	4	77	0
**RF-51**	CBD	0	2	1	85	0	0	2	0	238	0	2	14	2	89	1
**RF-52**	CBD	0	1	2	174	2	0	8	2	85	4	0	3	1	161	1
**RF-53**	CBD	0	8	0	47	1	0	0	0	79	1	0	6	4	210	12
**RF-54**	CBD	1	0	4	21	0	0	2	0	159	4	0	5	1	117	0
**RF-55**	CBD	0	4	3	77	3	1	1	1	31	0	1	6	1	113	3
**RF-56**	CBD	0	0	1	109	0	0	16	2	129	0	0	28	3	139	0
**RF-57**	CBD	0	1	7	91	0	3	10	3	116	0	1	12	1	156	0
**RF-58**	CBD	2	0	3	105	2	0	40	0	143	1	0	63	0	171	15
**RF-59**	CBD	0	0	4	95	0	0	6	0	154	4	0	10	6	222	5
**RF-60**	CBD	0	2	2	140	1	0	18	1	197	2	0	19	1	165	12
**RF-61**	CBD	0	12	3	178	2	0	1	0	30	11	0	12	3	188	1
**RF-62**	CBD	0	5	0	146	2	0	58	0	108	8	0	22	3	229	6
**RF-63**	CBD	0	0	5	78	0	0	1	5	182	0	0	3	5	185	2
**RF-64**	PiD	0	1	3	137	2	0	0	1	625	0	0	0	11	219	2
**RF-65**	PiD	0	0	6	153	2	0	0	3	281	0	0	0	2	290	1
**RF-66**	PiD	0	0	0	12	0	0	0	1	334	0	0	1	1	265	0
**RF-67**	PiD	0	0	0	183	0	0	0	0	198	0	0	0	0	320	0
**RF-68**	PiD	1	4	2	125	2	0	0	1	270	0	0	3	10	183	2
**RF-69**	PiD	0	0	1	121	0	0	0	4	320	0	2	2	6	67	1
**RF-70**	PiD	0	0	0	7	0	0	0	3	471	1	0	0	33	98	0
**RF-71**	PiD	0	0	2	77	0	0	0	1	457	0	0	0	4	299	0
**RF-72**	PiD	0	0	0	1	0	0	0	0	305	0	4	0	12	167	1
**RF-73**	PiD	0	0	0	1	0	0	0	1	79	0	0	0	0	56	1
**RF-74**	PiD	0	0	0	16	0	0	0	2	689	1	0	0	7	107	2
**RF-75**	PiD	0	0	0	20	0	0	1	1	269	0	0	0	1	293	3
**RF-76**	PiD	2	5	1	145	17	0	1	2	202	1	0	0	0	227	0
**RF-77**	PiD	0	0	0	4	0	0	0	1	437	1	0	1	5	233	1
**RF-78**	PiD	0	0	0	2	0	0	0	0	720	1	0	0	0	237	2
**RF-79**	PiD	0	0	2	148	6	0	0	2	239	0	0	0	0	218	5
**RF-80**	PiD	0	0	2	150	1	0	0	0	182	0	0	0	1	358	2
**RF-81**	PiD	0	1	1	3	0	0	0	0	670	0	0	0	1	223	0
**RF-82**	PiD	8	4	1	145	0	0	0	0	177	0	0	0	2	305	0
**RF-83**	PiD	0	0	1	71	0	1	0	0	306	1	0	1	0	170	3
**RF-84**	PiD	1	1	1	4	0	0	1	2	365	1	0	0	1	208	0
**RF-85**	PSP	69	7	15	49	3	59	2	7	10	0	56	0	12	15	0
**RF-86**	PSP	74	2	31	29	0	201	8	6	17	2	60	4	2	5	0
**RF-87**	PSP	3	0	13	16	3	29	5	4	15	2	0	0	3	1	0
**RF-88**	PSP	166	7	17	36	9	42	0	3	9	0	94	0	14	44	0

**RF-89**	PSP	19	0	18	31	16	31	0	6	34	0	3	7	2	60	7
**RF-90**	PSP	13	2	7	7	0	30	1	2	1	0	138	12	18	27	1
**RF-91**	PSP	5	4	0	9	13	15	5	3	21	4	0	0	1	108	22
**RF-92**	PSP	14	1	35	48	2	13	1	2	37	2	0	0	1	146	13
**RF-93**	PSP	17	1	70	24	0	40	0	13	20	0	10	1	16	18	1
**RF-94**	PSP	1	1	55	27	0	52	2	9	13	0	37	1	14	3	0
**RF-95**	PSP	4	0	3	2	0	20	1	7	6	0	4	0	3	5	0
**RF-96**	PSP	9	0	11	21	0	97	1	4	26	1	56	1	6	16	0
**RF-97**	PSP	12	0	24	91	23	12	0	7	24	0	0	2	3	93	16
**RF-98**	PSP	55	0	59	75	9	40	22	4	45	17	15	12	3	68	49
**RF-99**	PSP	36	0	39	63	1	112	9	0	35	0	21	0	5	14	0
**RF-100**	PSP	2	0	52	43	0	28	1	7	11	1	11	0	26	14	0
**RF-101**	PSP	19	0	51	13	0	66	1	8	16	0	6	0	16	9	0
**RF-102**	PSP	5	0	37	21	0	5	0	12	12	0	1	0	18	10	0
**RF-103**	PSP	6	2	50	34	0	61	4	8	17	0	0	0	1	3	0
**RF-104**	PSP	12	0	34	15	0	22	0	9	10	0	17	2	8	7	0
**RF-105**	PSP	52	0	42	33	0	114	0	6	6	0	27	0	20	5	0
**RF-106**	PSP	24	0	34	20	0	47	1	4	9	0	26	3	19	23	1
**RF-107**	PSP	33	0	103	61	1	99	0	18	20	1	71	1	63	57	1
**RF-108**	PSP	103	0	68	29	5	40	1	14	16	1	55	1	29	15	0
**RF-109**	PSP	3	0	14	21	0	11	0	2	25	0	11	2	3	10	0
**RF-110**	PSP	17	0	42	16	0	24	1	6	7	0	7	0	11	15	0
**RF-111**	PSP	28	0	46	26	0	65	3	3	22	0	22	0	13	21	0
**RF-112**	PSP	11	0	89	39	0	36	0	8	28	0	18	0	35	24	0
**RF-113**	PSP	4	0	24	30	0	77	1	12	10	0	28	2	32	17	0
**RF-114**	PSP	11	0	32	22	0	32	0	5	13	1	20	0	12	16	1
**RF-115**	PSP	9	0	28	16	0	116	1	6	8	0	15	1	15	7	0
**RF-116**	PSP	4	0	107	64	0	6	0	16	3	0	6	1	16	18	0
**RF-117**	PSP	10	0	50	42	0	109	1	11	9	0	17	1	12	13	0
**RF-118**	PSP	23	0	82	30	0	24	0	13	18	0	12	0	34	25	0
**RF-119**	PSP	32	0	67	47	0	12	1	11	29	0	25	0	16	13	0
**RF-120**	PSP	8	0	48	15	0	53	1	15	3	0	22	0	9	2	0

Abbreviations: AD, Alzheimer's disease; AP, astrocytic plaques; CB, coiled bodies; CBD, corticobasal degeneration; NI, neuronal inclusions; NP, neuritic plaques; PiD, Pick's disease; PSP, progressive supranuclear palsy; TA, tufted astrocytes.

Table S5: Quantitative burdens of cases in hold-out dataset (CP13) using Model 1

		Motor cortex					Caudate nucleus					Superior frontal gyrus				
**Case ID**	**Dx**	**TA**	**AP**	**CB**	**NI**	**NP**	**TA**	**AP**	**CB**	**NI**	**NP**	**TA**	**AP**	**CB**	**NI**	**NP**
HO-1	AD	0	0	3	74	19	0	0	0	14	0	0	0	8	147	4
HO-2	AD	0	0	1	66	17	0	0	0	2	0	1	0	0	45	1
HO-3	AD	1	0	1	128	8	0	0	0	15	0	0	0	0	100	1
HO-4	AD	2	0	2	12	16	1	0	0	20	1	0	0	0	139	16
HO-5	AD	0	0	0	57	3	0	0	0	16	1	0	0	0	170	12
HO-6	AD	1	1	0	43	49	0	1	0	7	0	0	0	0	128	16
HO-7	AD	0	1	2	33	46	0	0	0	11	0	0	0	0	120	4
HO-8	AD	0	0	0	81	30	0	0	1	17	0	0	0	0	174	5
HO-9	AD	0	0	0	1	2	0	0	0	7	0	0	2	1	54	3
HO-10	AD	0	0	0	30	32	4	2	0	16	0	0	0	0	153	12
HO-11	CBD	0	2	1	113	0	1	6	1	156	4	0	13	1	99	3
HO-12	CBD	1	14	1	43	0	0	8	1	144	0	0	74	1	60	0
HO-13	CBD	0	3	0	65	0	0	0	3	128	0	1	1	2	108	0
HO-14	CBD	0	4	0	43	0	0	4	1	191	0	0	3	2	128	0
HO-15	CBD	0	0	1	98	2	0	4	4	114	1	0	12	2	155	8
HO-16	CBD	0	2	0	104	0	0	0	0	57	0	0	0	4	213	4
HO-17	CBD	0	4	5	159	8	0	3	1	198	0	0	12	3	230	12
HO-18	CBD	0	5	3	170	16	0	12	1	160	1	0	23	5	189	25
HO-19	CBD	0	0	7	138	0	1	0	1	190	0	0	1	4	175	0
HO-20	CBD	0	1	2	83	3	0	8	1	217	5	0	10	2	95	6
HO-21	CBD	0	0	3	53	0	0	1	1	49	1	1	2	7	62	1
HO-22	CBD	0	1	7	43	0	0	21	3	123	1	1	7	5	57	1
HO-23	CBD	0	48	2	203	13	0	23	0	96	1	0	51	0	135	8
HO-24	PiD	0	0	2	46	0	0	0	0	139	0	0	0	2	189	0
HO-25	PiD	0	0	0	1	0	0	0	0	172	0	0	0	0	273	0
HO-26	PiD	0	0	0	4	0	0	0	2	636	0	0	0	20	154	1
HO-27	PiD	0	0	1	14	0	0	0	5	199	0	0	0	8	155	0
HO-28	PiD	0	0	0	0	0	0	0	7	264	0	2	1	2	245	2
HO-29	PiD	0	0	0	4	0	0	0	1	452	0	0	0	15	137	0
HO-30	PiD	8	0	1	17	0	0	0	1	493	0	0	0	6	318	1
HO-31	PiD	3	0	1	52	0	0	0	4	822	0	5	0	12	240	1
HO-32	PiD	3	0	0	47	0	0	0	0	443	7	0	0	4	171	0
HO-33	PiD	0	0	0	0	0	0	0	0	35	0	0	0	2	74	0
HO-34	PSP	9	0	58	49	1	104	4	14	27	0	45	0	31	25	1
HO-35	PSP	1	0	3	3	0	18	2	4	15	3	0	0	0	2	0
HO-36	PSP	3	0	34	37	0	20	0	3	12	0	30	0	19	24	0
HO-37	PSP	9	0	46	12	0	16	1	18	22	0	7	0	13	8	0
HO-38	PSP	146	1	28	25	0	4	1	1	1	0	91	4	21	18	0
HO-39	PSP	32	0	54	31	0	86	2	4	19	1	52	2	6	14	0
HO-40	PSP	15	0	32	17	0	107	3	6	18	1	46	0	47	26	0
HO-41	PSP	15	4	5	14	5	7	2	4	23	7	1	1	2	56	45
HO-42	PSP	0	0	14	10	0	0	0	0	7	0	0	0	0	2	0
HO-43	PSP	1	0	53	25	0	25	0	9	17	0	23	0	9	15	0
HO-44	PSP	29	1	29	14	0	0	0	1	21	0	9	0	14	20	0
HO-45	PSP	3	0	88	35	0	0	0	0	19	0	3	0	17	19	0
HO-46	PSP	75	0	48	79	0	9	0	3	7	0	5	0	6	26	2
HO-47	PSP	80	0	53	86	1	0	0	2	12	0	40	1	18	41	1
HO-48	PSP	11	0	47	28	1	37	3	3	17	0	10	0	10	11	0
HO-49	PSP	205	0	13	48	0	103	5	2	12	0	33	1	7	7	1
HO-50	PSP	3	3	14	39	56	3	1	3	38	7	0	0	1	112	33

Abbreviations: AD, Alzheimer's disease; AP, astrocytic plaques; CB, coiled bodies; CBD, corticobasal degeneration; NI, neuronal inclusions; NP, neuritic plaques; PiD, Pick's disease; PSP, progressive supranuclear palsy; TA, tufted astrocytes.

Table S6: Quantitative burdens of cases in hold-out dataset (AT8) using Model 1

		Motor cortex					Caudate nucleus					Superior frontal gyrus				
Case ID	Dx	TA	AP	CB	NI	NP	TA	AP	CB	NI	NP	TA	AP	CB	NI	NP
HO-1	AD	1	0	13	114	14	0	0	0	31	0	0	0	3	157	7
HO-2	AD	1	2	4	69	23	0	0	0	1	0	0	0	1	55	0
HO-3	AD	1	2	16	109	6	0	0	0	15	0	0	0	2	107	1
HO-4	AD	1	4	0	20	19	4	1	0	17	4	0	0	0	161	14
HO-5	AD	0	2	0	38	58	0	0	0	17	0	0	1	0	150	12
HO-6	AD	0	3	1	42	19	0	2	0	2	0	0	1	0	75	2
HO-7	AD	8	3	1	17	15	0	0	0	11	0	0	0	0	90	4
HO-8	AD	0	6	0	63	23	1	0	3	22	0	0	0	0	118	7
HO-9	AD	0	1	1	1	4	0	0	0	16	0	0	11	2	62	16
HO-10	AD	0	0	0	36	35	0	5	1	5	3	0	0	0	110	5
HO-11	CBD	0	2	0	112	1	0	6	0	129	6	8	17	5	79	1
HO-12	CBD	2	27	0	31	1	0	104	0	155	2	6	81	2	62	0
HO-13	CBD	0	2	1	56	0	0	25	0	133	11	0	4	1	70	0
HO-14	CBD	0	0	2	30	0	0	0	1	65	0	0	0	5	120	0
HO-15	CBD	0	0	10	136	1	0	7	3	155	0	0	4	1	126	6
HO-16	CBD	0	0	4	78	0	0	0	0	26	0	0	4	18	167	0
HO-17	CBD	0	11	9	182	11	0	18	0	179	0	0	39	4	234	4
HO-18	CBD	4	11	11	138	5	0	22	1	55	0	9	21	1	82	0
HO-19	CBD	1	0	2	120	1	0	5	0	196	1	0	11	0	133	1
HO-20	CBD	0	2	4	33	0	0	12	0	276	2	2	12	0	103	0
HO-21	CBD	0	0	5	58	0	0	2	0	28	0	0	0	1	73	0
HO-22	CBD	0	0	5	49	0	0	36	2	129	3	1	9	0	43	0
HO-23	CBD	0	1	3	92	0	0	9	1	59	0	0	11	4	145	0
HO-24	PiD	0	0	1	43	0	0	1	0	154	0	0	0	0	225	2
HO-25	PiD	0	0	0	1	0	0	0	1	113	0	0	1	1	133	8
HO-26	PiD	0	0	0	8	0	0	0	4	727	0	3	1	13	161	4
HO-27	PiD	0	1	2	91	0	0	4	14	411	0	4	1	10	268	3
HO-28	PiD	1	3	3	12	0	0	0	0	255	0	0	0	0	220	0
HO-29	PiD	0	0	0	8	1	0	0	1	655	2	0	3	11	138	0
HO-30	PiD	1	0	5	77	0	0	0	4	428	0	0	1	2	345	0
HO-31	PiD	5	1	2	49	0	0	0	3	770	0	13	8	7	241	3
HO-32	PiD	1	0	0	53	0	1	0	0	415	3	0	0	1	182	0
HO-33	PiD	0	0	0	0	0	0	0	3	147	0	0	0	1	172	2
HO-34	PSP	9	2	69	53	4	113	7	10	27	5	42	7	29	24	0
HO-35	PSP	2	0	3	4	0	10	1	0	12	0	0	0	0	3	0
HO-36	PSP	3	0	22	15	0	3	2	1	15	0	16	0	29	27	0
HO-37	PSP	12	1	43	12	0	15	2	4	19	0	9	2	29	13	0
HO-38	PSP	104	8	34	44	2	4	1	0	1	0	95	1	20	15	0
HO-39	PSP	18	0	32	32	0	58	2	6	20	2	26	0	8	26	0
HO-40	PSP	26	0	53	23	1	65	3	4	23	2	27	1	25	30	0
HO-41	PSP	21	0	6	12	1	12	0	1	19	2	0	1	1	37	29
HO-42	PSP	1	0	11	12	0	0	0	0	3	0	0	0	1	4	0
HO-43	PSP	3	0	18	26	0	17	0	2	19	0	13	1	4	22	0
HO-44	PSP	30	0	46	18	0	0	0	1	30	0	21	1	8	18	0
HO-45	PSP	14	0	27	31	0	0	0	1	27	0	12	1	15	18	0
HO-46	PSP	38	2	38	104	0	0	0	0	7	0	1	1	3	64	2
HO-47	PSP	10	0	52	55	1	0	0	0	3	0	39	1	23	49	1
HO-48	PSP	6	0	42	24	0	41	4	3	14	1	10	1	26	11	0
HO-49	PSP	107	1	17	34	0	37	1	4	12	0	21	0	8	5	0
HO-50	PSP	2	2	2	39	15	6	5	2	36	11	0	0	3	92	10

Abbreviations: AD, Alzheimer's disease; AP, astrocytic plaques; CB, coiled bodies; CBD, corticobasal degeneration; NI, neuronal inclusions; NP, neuritic plaques; PiD, Pick's disease; PSP, progressive supranuclear palsy; TA, tufted astrocytes.

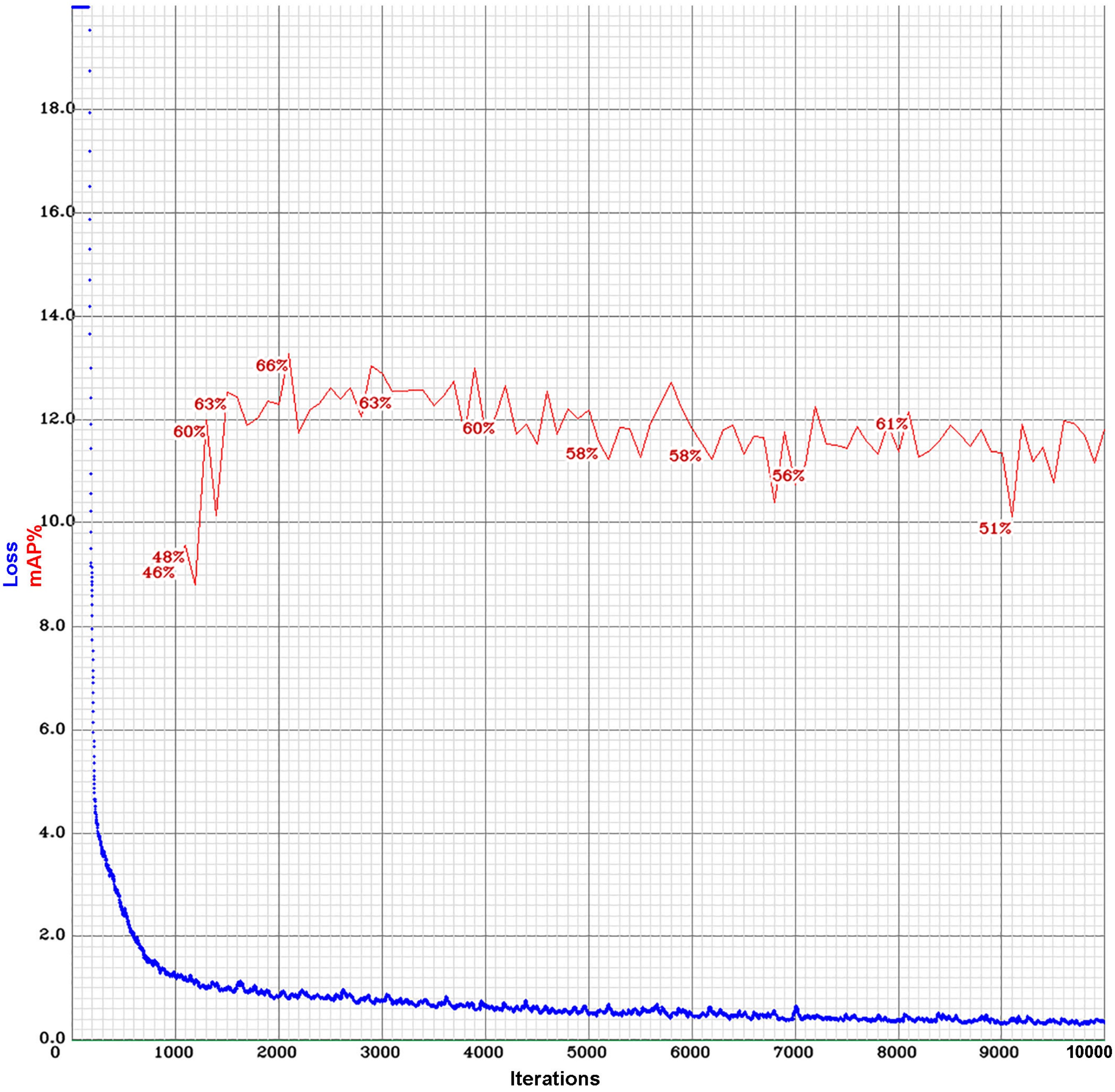

Figure S1: Training loss and mAP during training in Model 1

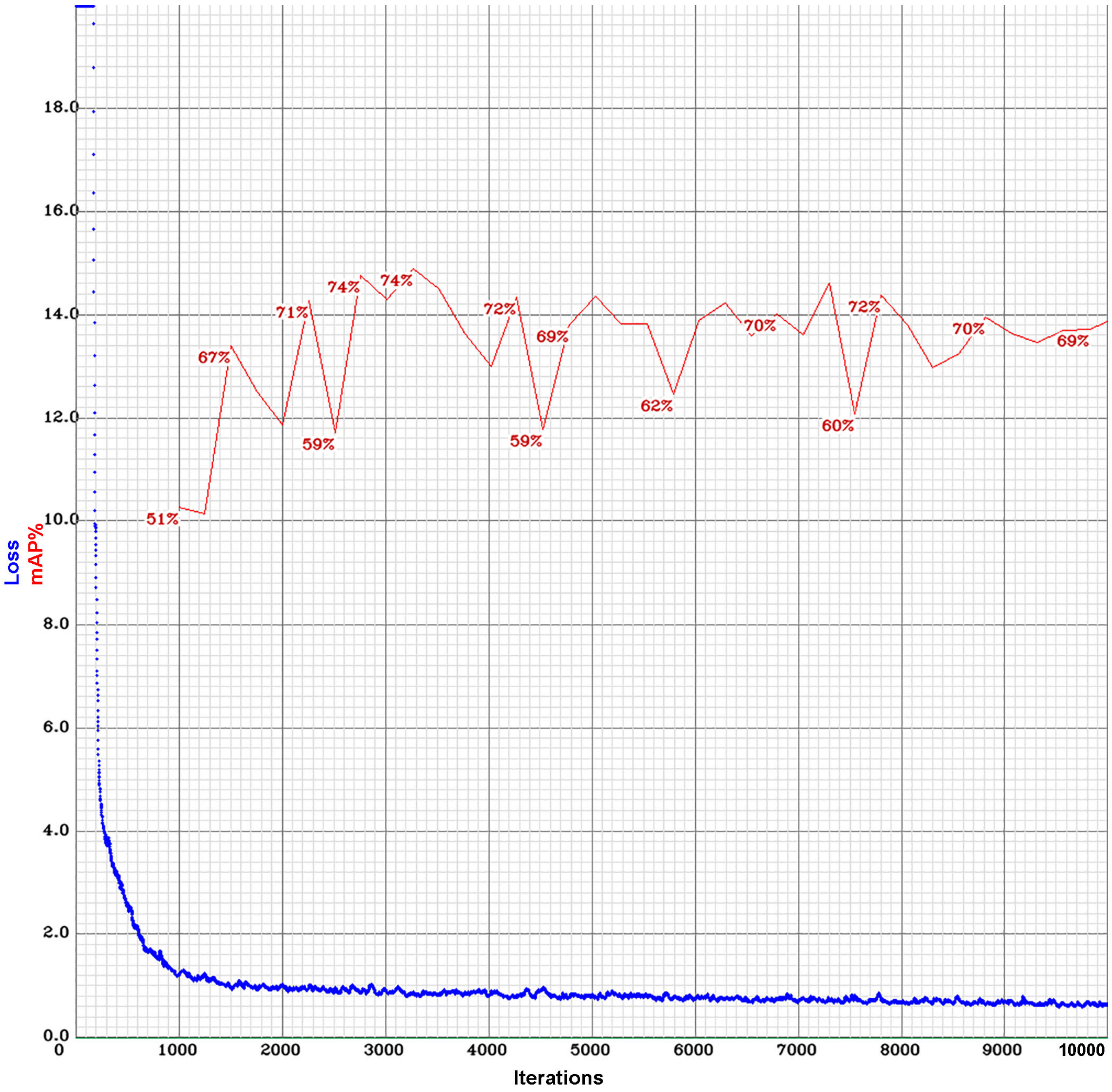

Figure S2: Training loss and mAP during training in Model 2

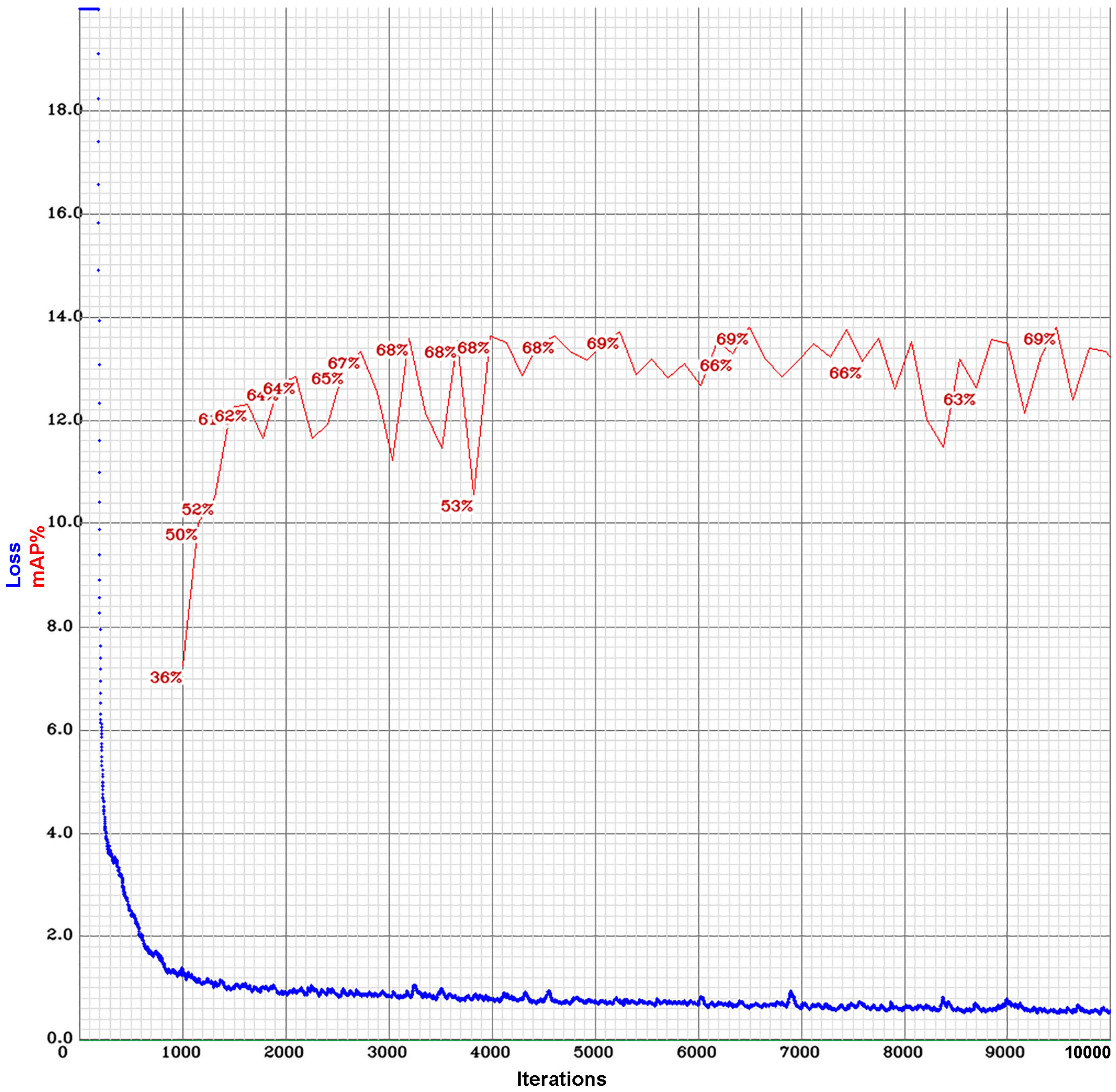

Figure S3: Training loss and mAP during training in Model 3

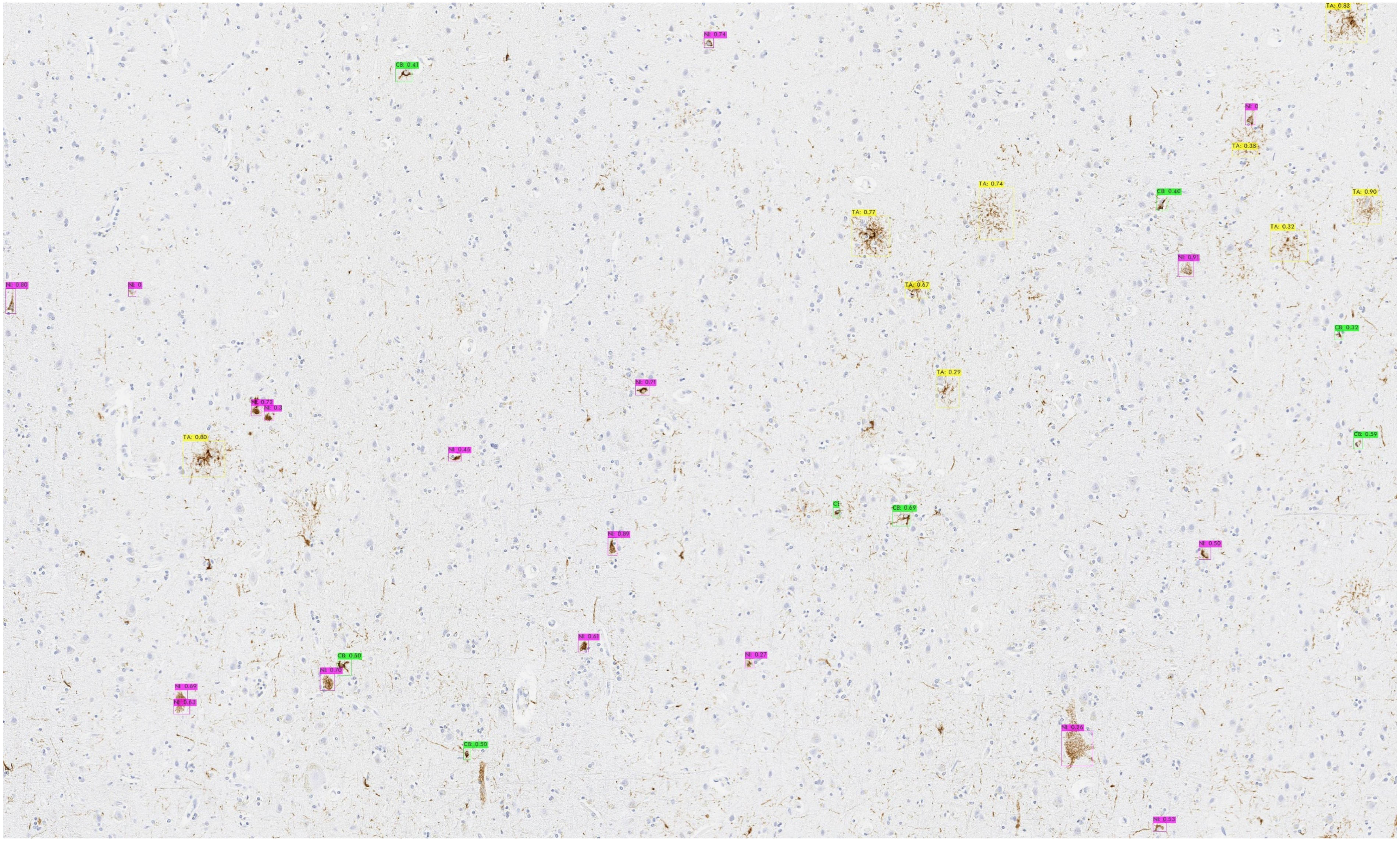

Figure S4: Representative image with bounding box. The motor cortex from a patient with PSP+AD (RF-96).

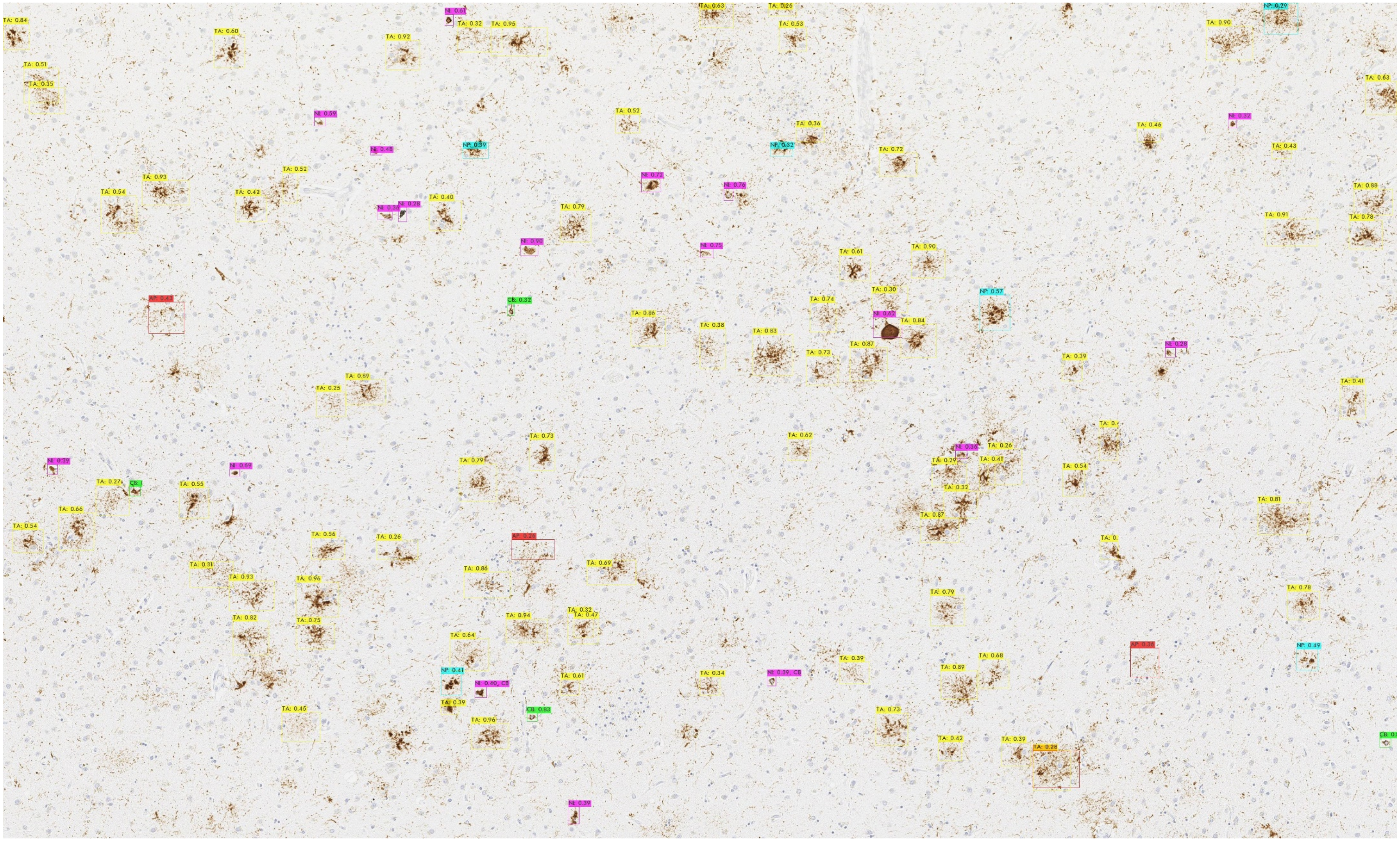

Figure S5: Representative image with bounding box. The caudate nucleus from a patient with PSP+AD (RF-96).

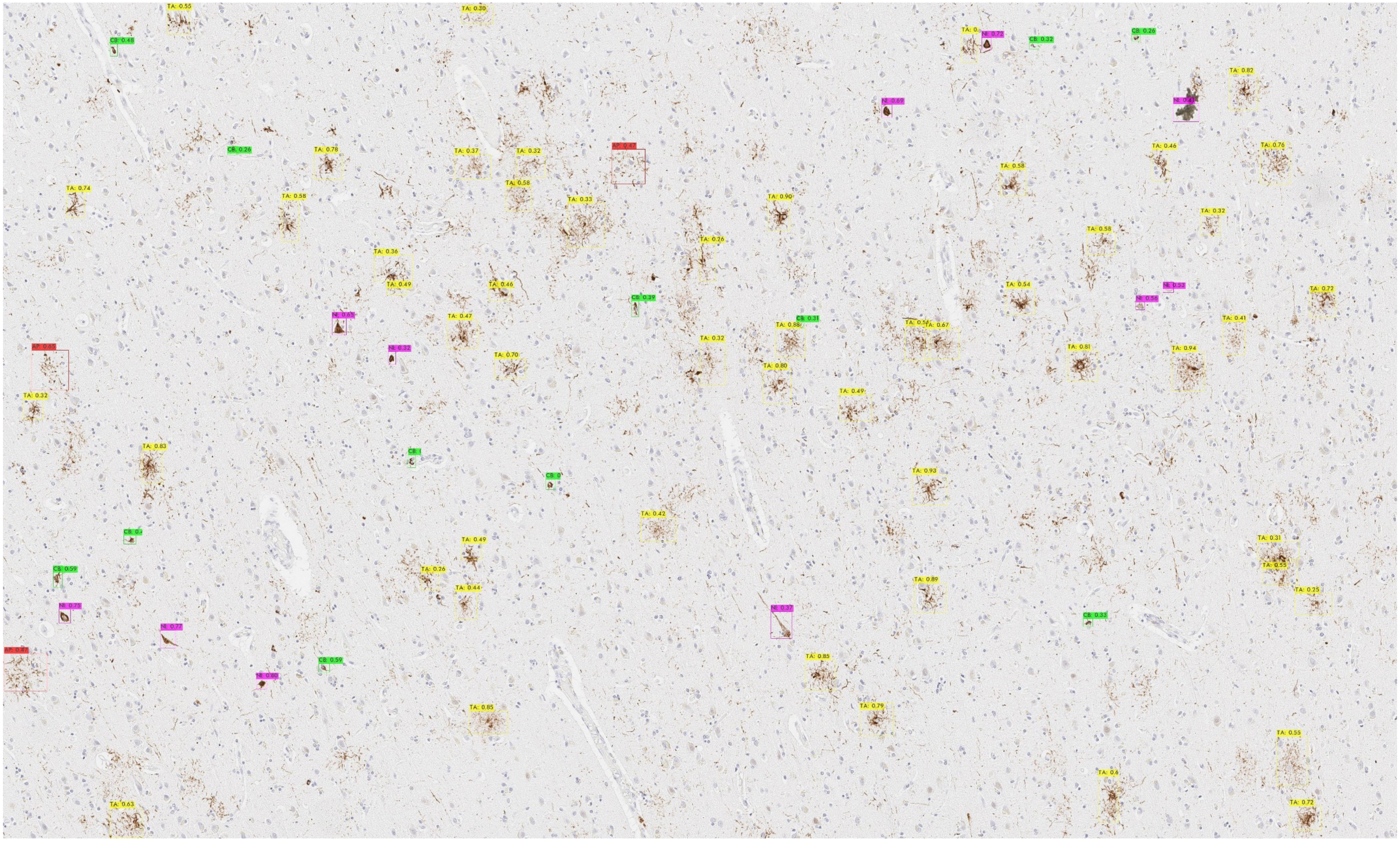

Figure S6: Representative image with bounding box. The superior frontal gyrus from a patient with PSP+AD (RF-96).

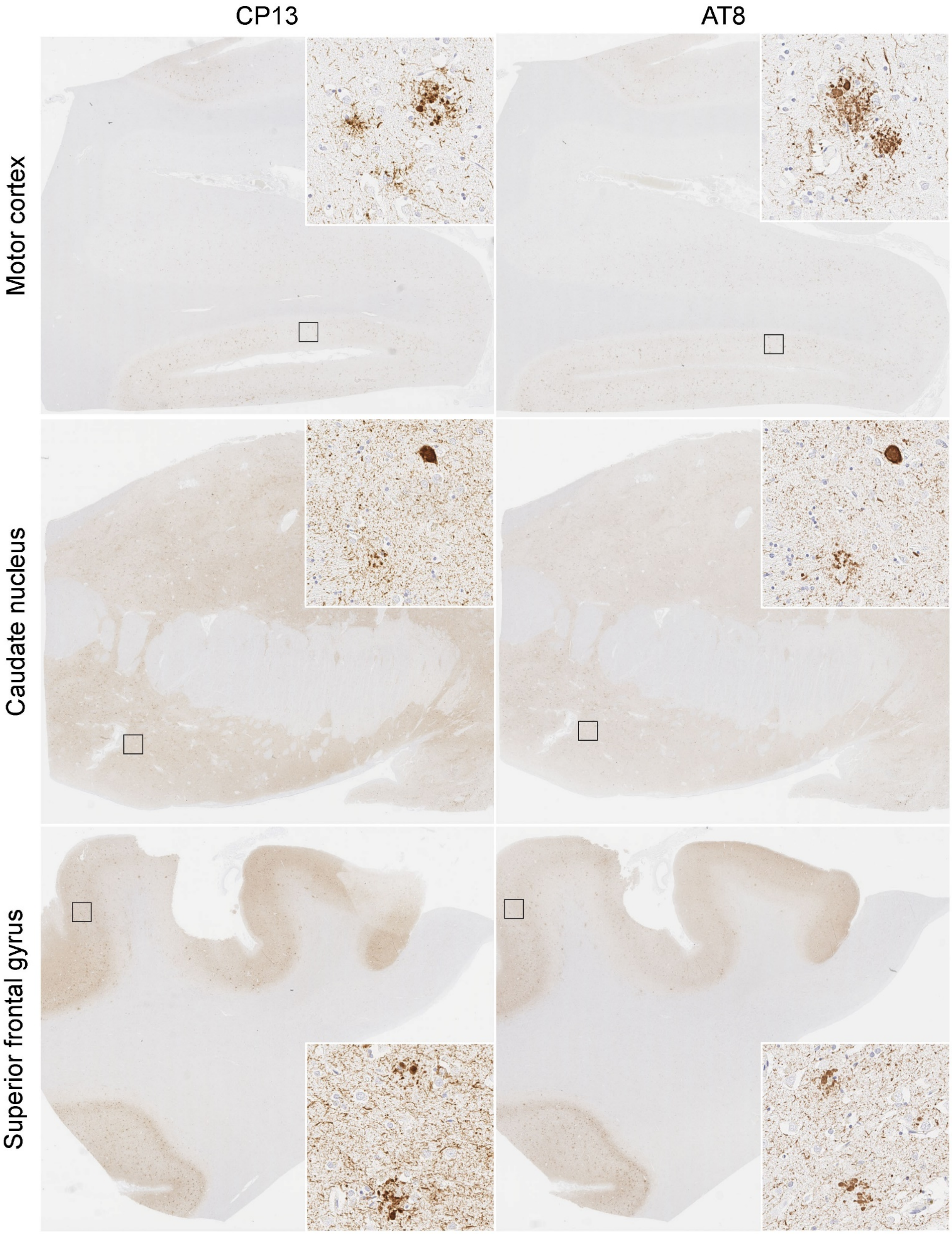

Figure S7: Comparison between CP13- and AT8-stained slides. Representative images are taken from a patient with PSP+AD (HO-41). Tufted astrocytes, neuritic plaques, and neuronal inclusions are present in the motor cortex, caudate nucleus, and superior frontal gyrus.
